# Deficiency of G9a boosts muscle regeneration through IL13/Musclin-mediated crosstalk between macrophage and myofiber

**DOI:** 10.1038/s41419-026-08944-2

**Published:** 2026-06-10

**Authors:** Ying Jin, Kaiyang Zhou, Siyu Hao, Hu Yue, Haoyu Wang, Zhiyuan Liu, Yihao Zhou, Yunhao Xie, Xinran Liu, Hong Chen, Yuxia Liu, Anlin Peng, Yangkai Li, Kun Huang, Ling Zheng

**Affiliations:** 1 State Key Laboratory of Metabolism and Regulation in Complex Organisms, TaiKang Center for Life and Medical Sciences; Frontier Science Center for Immunology and Metabolism, College of Life Sciences, Wuhan University, Wuhan, China; 2 School of Pharmacy, Tongji Medical College, Huazhong University of Science and Technology, Wuhan, China; 3 Division of Life Science, State Key Laboratory of Molecular Neuroscience, The Hong Kong University of Science and Technology, Hong Kong SAR, China; 4 Wuhan Third Hospital, Tongren Hospital of Wuhan University, Wuhan, China; 5 Department of Thoracic Surgery, Tongji Hospital, Tongji Medical College, Huazhong University of Science and Technology, Wuhan, China

**Keywords:** Physiology, Diseases

## Abstract

Muscle regenerative capacity declines with aging and disease, which leads to muscle loss and reduced lifespan. Muscle regenerative failure is related to a disrupted network orchestrated by multiple muscle-harbored cell types; whether and how the interplay between macrophages and myofibers contributes to this process is largely unknown. Herein, we report upregulation of histone methyltransferase G9a in both aged human muscle and mouse muscle after injury. Deletion of G9a in either myeloid cells or myofibers accelerates muscle regeneration. Mechanistically, G9a down-regulates macrophage-derived interleukin 13 (IL13) and suppresses myofiber-derived myokine musclin, respectively, to inhibit myogenesis and macrophage phenotype transition during muscle regeneration. Either IL13 or musclin, per se, accelerated muscle regeneration, and their combined administration showed synergistic effects with therapeutic potentials for muscle degeneration disorders. Collectively, we highlight a crosstalk between macrophages and myofibers through IL13-Stat6 signaling and musclin, both regulated by G9a, which steers a pro-recovery microenvironment after muscle injury, with therapeutic potentials for muscle degeneration disorders.

## Introduction

Skeletal muscle is an integral organ for physical movement and metabolism; emerging evidence demonstrates a positive correlation between the amount of muscle mass and lifespan [1]. More than a hundred million people are suffering from muscle degenerative disorders due to injury like trauma, or diseases such as aging-related sarcopenia and peripheral arterial disease [2–4]. Since most muscle degenerative disorders are accompanied by impaired muscle regeneration [5, 6], understanding how muscle regenerates after injury will facilitate identifying effective treatments for these disorders.

Muscle regeneration is a highly coordinated process regulated by multiple cell types. For examples, macrophages initiate inflammation response, muscle stem cells (MuSCs) drive myogenesis, fibro/adipogenic progenitors (FAPs) and endothelial cells assist tissue remodeling [7, 8]. Macrophages play critical roles during muscle regeneration. Upon injury, monocytes are recruited to injured muscle, develop into pro-inflammatory (Ly6C^high^) macrophages and initiate a cascade of events [9]. Next, the pro-inflammatory macrophages secret several cytokines to promote the activation and proliferation of MuSCs, and clear injury-induced cell debris by phagocytosis during inflammatory phase. Subsequently, pro-inflammatory macrophages transit to anti-inflammatory (Ly6C^low^) ones which triggers the resolution of inflammation, while any delay in this transition results in regeneration failure [10]. In the subsequent restorative phase, macrophages secret abundant cytokines including insulin-like growth factor 1 (IGF-1), transforming growth factor-β (TGF-β), and growth differentiation factor 3 (GDF-3), which act on MuSCs and other muscle cell types including endothelial cells and FAPs to stimulate myogenesis and muscle remodeling [9, 11]. Besides macrophages, myofibers, where MuSCs reside, also contribute to muscle regeneration by secreting myokines, including dickkopf 3 (DKK3), myostatin and interleukin 6 (IL6), which target MuSCs or T helper cells [12–14]. Therefore, the skeletal muscle microenvironment is highly dynamic during injury-induced regeneration, and well-orchestrated interplay among various muscle cell types steers the process, which is mediated by abundant secreted factors. Macrophages and myofibers, serving as sources of cytokines and myokines, represent promising targets for muscle regeneration.

Accumulating evidence suggests that altered histone modifications like methylation are associated with muscle regeneration [15]. G9A, encoded by *EHMT2*, is an epigenetic enzyme that catalyzes mono- and di-methylation of lysine 9 of histone H3 (H3K9me1/me2) [16]. H3K9me1 is generally linked to transcriptional activation, whereas H3K9me2 usually acts as a hallmark of transcriptional repression [17], and G9A preferentially catalyzes H3K9me2 in cultured cells [18]. G9A has been reported to play critical roles in myogenesis both in vivo and in vitro. Globally inhibiting G9a by its inhibitor A366 facilitates muscle regeneration through driving FAPs to execute the myogenic differentiation in mouse models [19]. Moreover, G9a has been reported to inhibit myoblast differentiation through the methylation of MyoD in vitro [20]. However, deletion of G9a in myoblasts (driven by MyoD-Cre recombinase) or MuSCs (driven by Pax7-Cre recombinase) in mouse shows no obvious effect on muscle development, satellite cells proliferation and differentiation, as well as muscle regeneration [21], suggesting that G9a may be involved in regeneration in other specific cell types, such as myofiber and/or macrophage, within muscle.

Here, we report elevated expression of G9A in aged human muscle, as well as in two mouse models of muscle regeneration, cardiotoxin (CTX) or hindlimb ischemia (HLI) induced muscle injury, especially in macrophages and myofibers. Specific deletion of G9a in myeloid cells (*Ehmt2*^*Lyz2*^ KO) or myofibers (*Ehmt2*^*HSA*^ KO) accelerates muscle regeneration. Furthermore, deletion of G9a in myeloid cells facilitates muscle regeneration through macrophage-derived IL13 to improve myogenesis, and augments IL13-Stat6 signaling to promote macrophage phenotype transition (pro-inflammatory to anti-inflammatory type); while G9a ablation in myofibers facilitates regeneration by myofiber-derived musclin that also stimulates myogenesis and macrophage phenotype transition. Either IL13 or musclin treatment accelerates muscle regeneration, and their combination further boosts regeneration, which serves as a feasible strategy for muscle degenerative disorders.

## Materials and methods

### Animals

*Ehmt2*^*flox/flox*^ mice in the C57BL/6 background were intercrossed with *Lyz2*-Cre mice (both from GemPharmatech, Nanjing, China) to generate myeloid cell-specific knockout (*Ehmt2*^*Lyz2*^ KO) mice. Myofiber-specific G9a knockout (*Ehmt2*^*HSA*^ KO) mice were generated as we previously reported [22]. Genotyping was performed with primers listed in Table S1. *Ehmt2*^*flox/flox*^; Lyz2-Cre^+^ (*Ehmt2*^*Lyz2*^) or *Ehmt2*^*flox/flox*^; HSA-Cre^+^ (*Ehmt2*^*HSA*^) and their respective littermates without Cre recombinase were used as cell type-specific knockout (KO) and wildtype (WT) mice. 18-month-old mice were used for aging experiments, and 2-3-month-old mice were used for muscle regeneration experiments.

To generate *Ehmt2*^*HSA*^; *Ostn*^*−/−*^ (DKO) mice, *Ostn*^*−/−*^ mice in C57BL/6 background (obtained from GemPharmatech) were intercrossed with *Ehmt2*^*HSA*^ mice to generate *Ehmt2*^*flox/+*^; HSA-Cre^+^; *Ostn*^*+/−*^ and *Ehmt2*^*flox/+*^; HSA-Cre^-^; *Ostn*^*+/−*^ mice. Then *Ehmt2*^*flox/+*^; HSA-Cre^+^; *Ostn*^*+/−*^ mice were further intercrossed with *Ehmt2*^*flox/+*^; HSA-Cre^-^; *Ostn*^*+/−*^ mice to generate *Ehmt2*^*flox/flox*^; HSA-Cre^+^; *Ostn*^*+/−*^ and *Ehmt2*^*flox/flox*^; HSA-Cre^-^; *Ostn*^*+/−*^ mice, which were intercrossed to generate *Ehmt2* ^*flox/flox*^; HSA-Cre^+^; *Ostn*^*−/−*^ (DKO: *Ehmt2*^*HSA*^; *Ostn*^*−/−*^), *Ehmt2*^*flox/flox*^; HSA-Cre^+^; *Ostn*^*+/+*^ (myofiber-specific G9a KO: *Ehmt2*^*HSA*^; *Ostn*^*+/+*^), *Ehmt2*^*flox/flox*^; HSA-Cre^-^; *Ostn*^*−/−*^ (*Ostn* KO: *Ehmt2*^*flox/flox*^; *Ostn*^*−/−*^) and *Ehmt2*^*flox/flox*^; HSA-Cre^-^; *Ostn*^*+/+*^ (WT control) mice for double knockout (DKO) experiments.

C57BL/6 (2-3-month-old) mice were purchased from Hubei Provincial Center for Disease Control and Prevention for time-course experiments of muscle regeneration and for drug treatments. All mice were maintained in a specific-pathogen-free, temperature-controlled (22 °C ± 1 °C) and humidity-controlled (55 ± 5%) animal facility with a 12-h light/dark cycle and free access to water and food (Huafukang Bio., Beijing, China). Animal studies were approved by the Committee on Ethics in the Care and Use of Laboratory Animals of the College of Life Sciences, Wuhan University (WDSKY0201705-2) and handled according to the Guidelines of the China Animal Welfare Legislation. Male mice were used in this study. We selected sample sizes based on our experience. Mice were assigned to experimental groups based on their genotypes and were further randomly assigned to treatment or non-treatment groups; after assignment, no animal was excluded from our studies.

### Muscle injury models

Cardiotoxin (CTX) was used to induce skeletal muscle injury as previously described [14]. Briefly, mice were anesthetized with isoflurane inhalation, and 50 μL of 1 mg/mL CTX (in sterile saline) (Boyao Biological, Shanghai, China) was injected into the tibialis anterior (TA) muscle. Hindlimb ischemia (HLI) was also performed as described [23]. Briefly, after anesthetization, hair from the hindlimb to the midriff was shaved, and the skin was sterilized. After skin incision, the femoral artery was ligated and removed as long as the attached side-branches. Mice were sacrificed, and TA muscles were collected for experiments at the indicated days after either injury.

### In vivo drug administration

Mouse IL13 or a STAT6 inhibitor AS1517499 (both from MedChemExpress, Monmouth Junction, NJ) was dissolved in sterile saline or 10% DMSO plus 90% corn oil, respectively. 50 μL of IL13 (5 μg/mL) or saline was intramuscular injected into TA muscle once every two days after muscle injury. AS1517499 (10 mg/kg) was intraperitoneally injected one h before muscle injury, and every two days after muscle injury. Mouse Musclin-F and Musclin-33 were expressed or synthesized as we previously reported [22]. Mice were subcutaneously injected with 50 μL of 0.25 μg/μL Musclin-F or Musclin-33 or sterile saline twice per day after muscle injury.

For combined treatment, 50 μL of IL13 (2.5 μg/mL) was intramuscularly injected into TA muscle every two days, together with 50 μL of Musclin-F (0.25 μg/μL) subcutaneously injected once a day; while, the same dosage for each drug was used as the single drug treatment controls.

### In situ muscle tetanic contraction force measurement

In situ muscle tetanic contraction force was measured by an Aurora Scientific 1300A Whole Animal System (Aurora, ON, Canada) as previously described [24]. Briefly, at Day 7 after CTX-injury, mice were anaesthetized and the TA muscle was detached from tibia. Two platinum electrodes were placed between the TA muscle and the tibia, then fixed with the electrode holder. The optimal muscle length (L_0_) was determined by a series of 0.4 Hz and 0.01 Hz stimulation respectively that elicited the maximal force. The muscle was stimulated at 10 mA, and the stimulus frequency was from 10 to 160 Hz (10, 20, 40, 50, 60, 80, 100, 120, 140 and 160 Hz). Muscle forces were normalized to muscle weight to generate the relative tetanic contraction force.

### Treadmill exercises

18-month-old mice received adaptive training as we previously reported [24]. During the test, mice first warmed up at 5 m/min, -5°downhill for 10 min, then the treadmill speed was gradually increased to 20 m/min, the mice kept running on the treadmill at 20 m/min until exhausted. Total running distance from start to exhaustion was recorded for the first run, and after one day of rest, for the second run.

### Human tissue specimens

Collection and use of human muscle samples were approved by the Wuhan Third Hospital (KY2023-027) and Tongji Hospital of Tongji Medical College (TJ-IRB202408023). The study was performed according to the Declaration of Helsinki, and informed consent was obtained. Muscle samples were collected from the site of patients with muscle injury caused by electrothermal burn or thoracoscopic port insertion in patients with lung cancer during lung resection surgery.

### Cell culture and treatments

THP-1 cells and L-929 cells (as the source of macrophage colony-stimulating factor (M-CSF)) are kind gifts from Dr Bo Zhong (Wuhan University), while C2C12 cells were obtained from Cell Bank/Stem Cell Bank (Chinese Academy of Sciences, Shanghai, China). THP-1 cells were cultured in RPMI-1640 medium (Gibco, Grand Island, NY) plus 10% FBS (Lonsera, Shanghai, China). L-929 cells and C2C12 cells were cultured in growth medium (DMEM (Gibco) plus 10% FBS (Lonsera)) to proliferate. STR profiling was provided by the providers, and mycoplasma contamination has been tested routinely. For C2C12 cells differentiation, the growth medium was replaced by differentiation medium (DMEM plus 2% Hyclone horse serum) and cultured for 3 more days. For drug treatments, 10 ng/mL IL13, or 3 ng/mL Musclin-F or Musclin-33 was added into the differentiation medium and replenished every two days.

### Bone marrow-derived macrophages (BMDMs) isolation, polarization and treatments

BMDMs were isolated as previously described [25]. Briefly, BMDMs were flushed out from the femur and tibia of mice, and red blood cell lysis buffer (Beyotime Biotech., China) was used to remove red blood cells. After filtering and centrifuging, the cell pellets (BMDMs) were cultured with RPMI-1640 medium plus 10% FBS and 20% conditioned medium collected from L-929 cells as the source of M-CSF for three days. On the fourth day (Day 4), BMDMs are regarded as macrophages [26] and cultured under RPMI-1640 medium plus 10% FBS, and are ready for different polarization stimulation and/or treatments.

To induce different polarization, BMDMs were treated with 50 ng/mL lipopolysaccharides (LPS) (Sigma, Saint Louis, MO) plus 30 ng/mL interferon-γ (IFN-γ; PeproTech, Cranbury, NJ) or 20 ng/mL IL4 (PeproTech) for 24 h to induce M1-like or M2-like macrophages, respectively. To inhibit the enzyme activity of G9a on polarization, BMDMs were treated with 2 μM BIX or 1 μM A366 (both from TargetMol, Shanghai, China) for 12 h, or transfected with either plasmid (pCAGGS, pCAGGS-G9a, pCAGGS-G9aΔSET) (as we previously constructed [27]) using Lipofectamine 3000 (Invitrogen, Carlsbad, CA), before stimulation of different polarization. To investigate the effect of Musclin-F or Musclin-33 on polarization, BMDMs were treated with 3 ng/mL Musclin-F or Musclin-33 or PBS for 24 h, before stimulation of different polarization.

To activate Stat6, BMDMs were starved with serum-free medium for 12 h, then treated with 20 ng/mL IL4 or IL13 for 20 min.

### Co-culture experiments

To obtain Musclin-treated or non-treated conditioned medium (CM) from BMDMs, BMDMs were treated with or without 3 ng/mL Musclin for 6 h on Day 4, then replaced with fresh medium, and the CM from BMDMs was collected 24 h later. To obtain IL13-treated or non-treated CM from C2C12 cells, C2C12 cells were treated with 20 ng/mL IL13 for 6 h on Day 4, then replaced with fresh medium, and the CM from C2C12 cells was collected 24 h later. Next, C2C12 cells were incubated with a 1:1 ratio of CM from BMDMs and C2C12 differentiation medium for 72 h, while BMDMs were treated with a 1:1 ratio of CM from C2C12 cells and M1-like or M2-like macrophage polarization medium for 24 h.

### Flow cytometry analysis

TA muscles were minced and digested at 37 °C in DMEM containing 2 mg/mL collagenase D (Sigma) and 0.5 U/mL Dispase II (Roche, Mannheim, Germany) for 1.5 h. After being treated with red blood cell lysis buffer, cells were filtered, centrifuged, and resuspended to obtain a single-cell suspension, which was incubated with anti-CD16/CD32 antibodies (BD, Bergan, NJ) for 10 min to block FcγRⅢ/Ⅱ, and then stained with PE-F4/80 and APC-Ly6C for 30 min at 4 °C (listed in Table S2). The cell suspension was further analyzed with a Cytoflex LX flow cytometer (Beckman Coulter, Brea, CA) and the data were analyzed with FlowJo analysis software (version 9.2). Gating was done as follows: integral cells (SSC-A (Side Scatter-Area), FSC-A (Forward Scatter-Area)), single cells (FSC-H (Forward Scatter-Height), FSC-A), live and dead cells (DAPI), macrophages (F4/80^+^), pro-inflammatory or anti-inflammatory cells (Ly6C^high^ or Ly6C^low^).

### IL13 and Musclin measurements

The levels of IL13 in TA muscle and medium were determined by a mouse IL13 ELISA kit (Elabscience, Wuhan, China), while the levels of Musclin in medium were determined by a mouse Musclin ELISA kit (Cusabio, Wuhan, China).

### Western blots and quantitative real-time PCR (qPCR)

Western blots and quantitative real-time PCR (qPCR) were performed as previously described [28]. Primers and antibodies used in the present study are provided in Tables S1 and S2. Densitometric quantification of Western blots was performed using Quantity One (Bio-Rad). The densitometric values of targeted proteins were normalized to those of the corresponding reference protein, then further normalized to the control group which was set as one, and reported as fold changes.

### Chromatin immunoprecipitation (ChIP) assay

The ChIP assay was performed as previously described [29]. Briefly, THP-1 cells were fixed with 1% formaldehyde and quenched with glycine. Crosslinked chromatin was sheared into 200-1000 bp. Chromatin was immunoprecipitated with an anti-G9a antibody (Table S2). DNA was isolated and detected by qPCR (primers listed in Table S1), and input samples were used as the internal control.

### Dual-luciferase reporter assays

Luciferase reporter assays were performed as we previously described [30]. Briefly, the *STAT6* promoter from –1000 bp to TSS (transcription start site) was cloned into pGL3-enhancer. THP-1 cells were co-transfected with pGL3-enhancer-STAT6, pRK-G9a, and pRL-TK Renilla luciferase reporter plasmid. After 24 h, cells were lysed, and firefly luciferase activity was detected.

### Histology, immunohistochemical and immunofluorescent staining

Histology, immunofluorescent staining of the TA muscles and cells, was performed as we previously described [24]. For pathological analysis, TA muscles were stained with Hematoxylin and Eosin (H&E), and the muscle cross-sectional area (CSA) was manually counted with Image-Pro Plus 6.0. For immunofluorescent staining, TA muscles or cells were incubated with indicated primary antibody (Table S2) at 4 °C overnight, then Alexa Fluor 488- or 594-conjugated secondary antibody (Table S2) was added for 2 h at room temperature. Images were taken with a Leica TCS SP8 confocal microscope (Leica, Germany). Quantitative analysis was analyzed by manual counting using Image-Pro Plus 6.0 software.

### Myotube fusion index

Fusion index was analyzed as previously described [31]. Fusion index = the number of nuclei in MyHC-positive cells with two or more nuclei * 100% / the number of total nuclei.

### Evaluation of the synergism between IL13 and Musclin

The relative fold change of myofiber CSA between single treatment group and combined treatment group was used to evaluate the synergism between IL13 and musclin. The modified probability sum test was used with the formula: q = P_IL13+musclin_/ (P_IL13_+P_musclin_- P_IL13_*P_musclin_). q < 0.85 indicates antagonistic, and q > 1.15 indicates synergic.

### RNA-sequencing (RNA-seq) and data analysis

Total RNA from the TA muscle of WT and *Ehmt2*^*HSA*^ KO mice was isolated. Equal amounts of RNA (*n* = 6) from the same group were combined into two samples for RNA-seq. Sequencing and data analysis were performed as we previously described [24]. RNA-Seq and single-cell RNA sequencing (scRNA-seq) data were downloaded from GEO (PRJNA509121 and GSE138826, respectively) [32, 33]. Quality control was performed using fastp (v0.24.0), followed by read alignment to the human reference genome (GRCh38, Ensembl release-104) and transcriptome (GRCh38, Ensembl release-104) using STAR (v2.7.11b). Gene expression quantification was conducted with RSEM (v1.3.1). Expression data for genes of interest were extracted from the gene expression matrix and compared across different groups. To facilitate cross-sample comparisons of gene expression levels, we utilized transcripts per million (TPM) as the expression metric.

### Statistical analysis

All the data were expressed as the mean ± SD (standard deviation). All statistical analyses were performed using GraphPad Prism 9 (San Diego, CA). Statistical significance was analyzed with the nonparametric Mann-Whitney test for comparison of two groups or with the Kruskal-Wallis test followed by the Mann-Whitney test for comparison of multiple groups. *P* < 0.05 was considered statistically significant. *n* was defined as the number of mice per group for in vivo studies, while *n* represented biological replicates for in vitro studies (at least three independent experiments were performed). The *n* number and statistical methods used for each analysis were indicated in the figure legends.

## Results

### G9a is involved in age-related muscle dysfunction

**Fig. 1:**
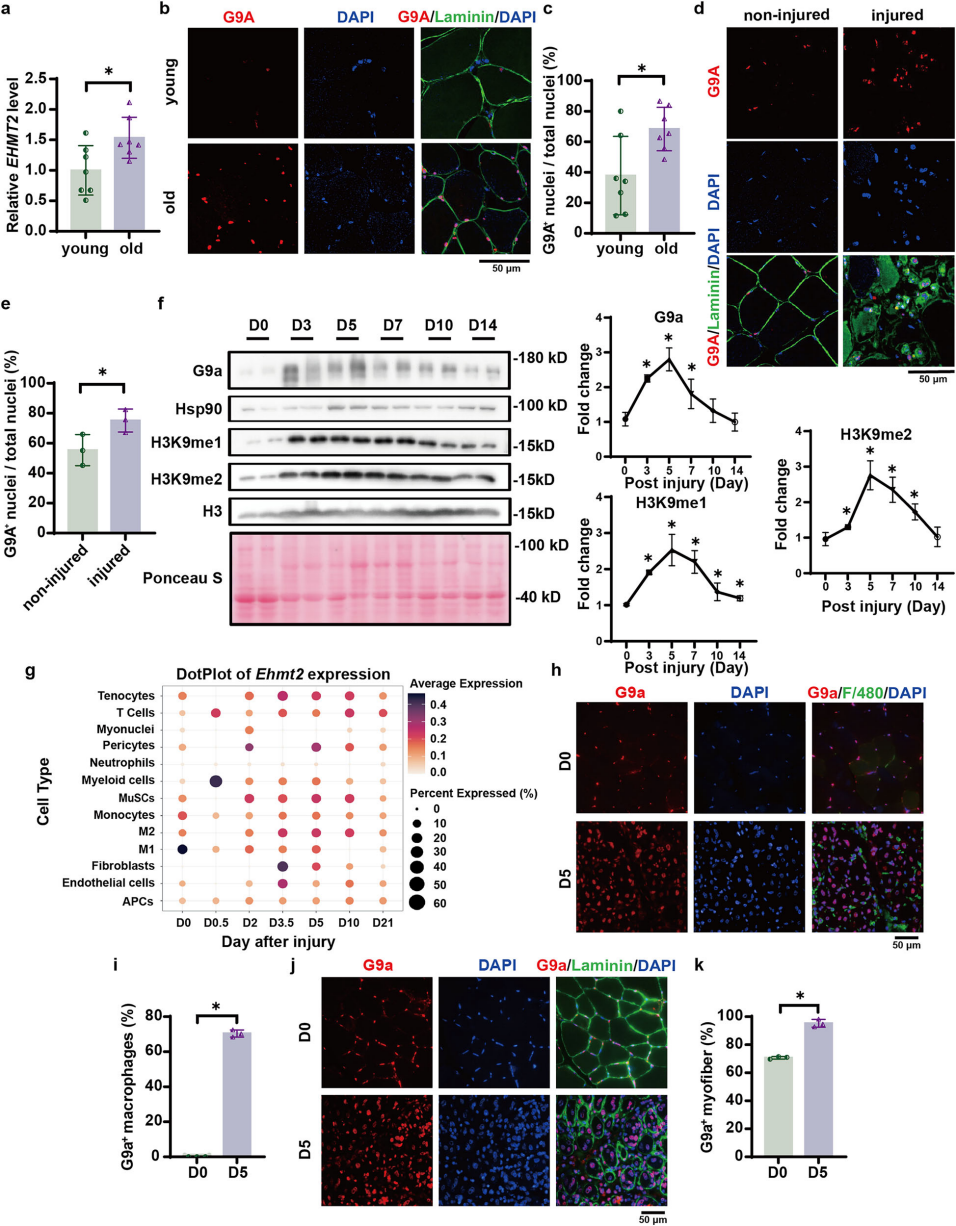
G9a is dynamically altered during muscle regeneration. **a** *EHMT2* expression levels in the vastus lateralis muscle of young or aged human subjects obtained from a published RNA-seq database. Representative co-immunostaining pictures of G9A and Laminin (**b**) with quantitative analysis (**c**) in the muscle of young and aged human. Representative co-immunostaining pictures of G9A and Laminin (**d**) with quantitative analysis (**e**) in the muscle of aged human with or without electrothermal burn. Red, G9a staining; green, Laminin staining; blue, DAPI stained nuclei for (**b, d**). **f** Western blots (left) with quantitative analysis (right) of G9a (normalized to Hsp90) and H3K9me1/2 (normalized to histone H3) in the TA muscle of C57BL/6 mice at indicated times after CTX-injury. Ponceau S staining was used as an additional loading control to confirm equal protein loading. **g** *Ehmt2* expression levels in the TA muscle of C57BL/6 mice at indicated times after CTX-injury (obtained from a published scRNA-seq database). Representative co-immunostaining pictures of G9a and F4/80 (**h**) or and Laminin (**j**) with their respective quantitative analysis (**i,** **k**) in the TA muscle of C57BL/6 mice at Day 0 and 5 after CTX- injury. Red, G9a staining; green, F4/80 or Laminin staining; blue, DAPI-stained nuclei for (**h, j**). TA tibialis anterior, CTX cardiotoxin. *n* = 3-7 per group. Data shown as mean ± SD. The Mann-Whitney test was used for statistical analysis. *P < 0.05.

Epigenetic modifications are altered during aging [34]. A significant increase in the transcriptional levels of *EHMT2*, the gene encoding histone methyltransferase G9A, was found in skeletal muscle of senior subjects compared with young subjects by analyzing a published RNA-seq data [32] (Fig. 1a). Compared with young human muscles, elevated protein levels of G9A were found in aged human muscles (Fig. 1b, c). Moreover, elevated levels of G9A were also found in aged human muscles after injury compared with non-injured muscles (Fig. 1d, e), indicating G9A may contribute to muscle regenerative capacity.

To investigate whether G9A is also involved in muscle regeneration, a classic CTX injection mouse model, and a mouse model mimic pathophysiological muscle injury conditions during organ-transplantation surgery, HLI, were used. After CTX injection, G9a protein level was dramatically increased and peaked at Day 5, then gradually declined while still higher than normal until Day 14 (Fig. 1f). In the HLI-induced muscle injury model, it was increased and peaked at Day 7, then decreased while still higher than normal until Day 12 (Fig. S1). The H3K9me1 and H3K9me2 levels were accordingly changed in these muscle injury models (Figs. 1f and S1).

Muscle regeneration is a precisely orchestrated process involving multiple cell types within muscle [7]. To investigate in which cell type G9a responds to muscle injury, a published single-cell RNA-seq of CTX-induced mouse muscle regeneration was analyzed [33], in which *Ehmt2* levels were most markedly elevated at Day 0.5 in myeloid cells and declined after Day 2, but remained higher than normal until Day 21 (Fig. 1g). Our co-immunostaining further confirmed dramatically elevated G9a levels in macrophages at Day 5 after CTX-injury (Fig. 1h, i). Single-cell RNA sequencing is not appropriate to analyze gene expression patterns in myotube/myofibers because they are giant multinucleated cells [35], we then co-stained G9a with myofiber, and found that elevated G9a levels in myofiber were also found at Day 5 after CTX-injury (Fig. 1j, k). These data show dynamically up-regulated G9a, at least in macrophages and myofibers, during muscle regeneration.

### Loss of G9a in myeloid cells accelerates muscle regeneration

**Fig. 2:**
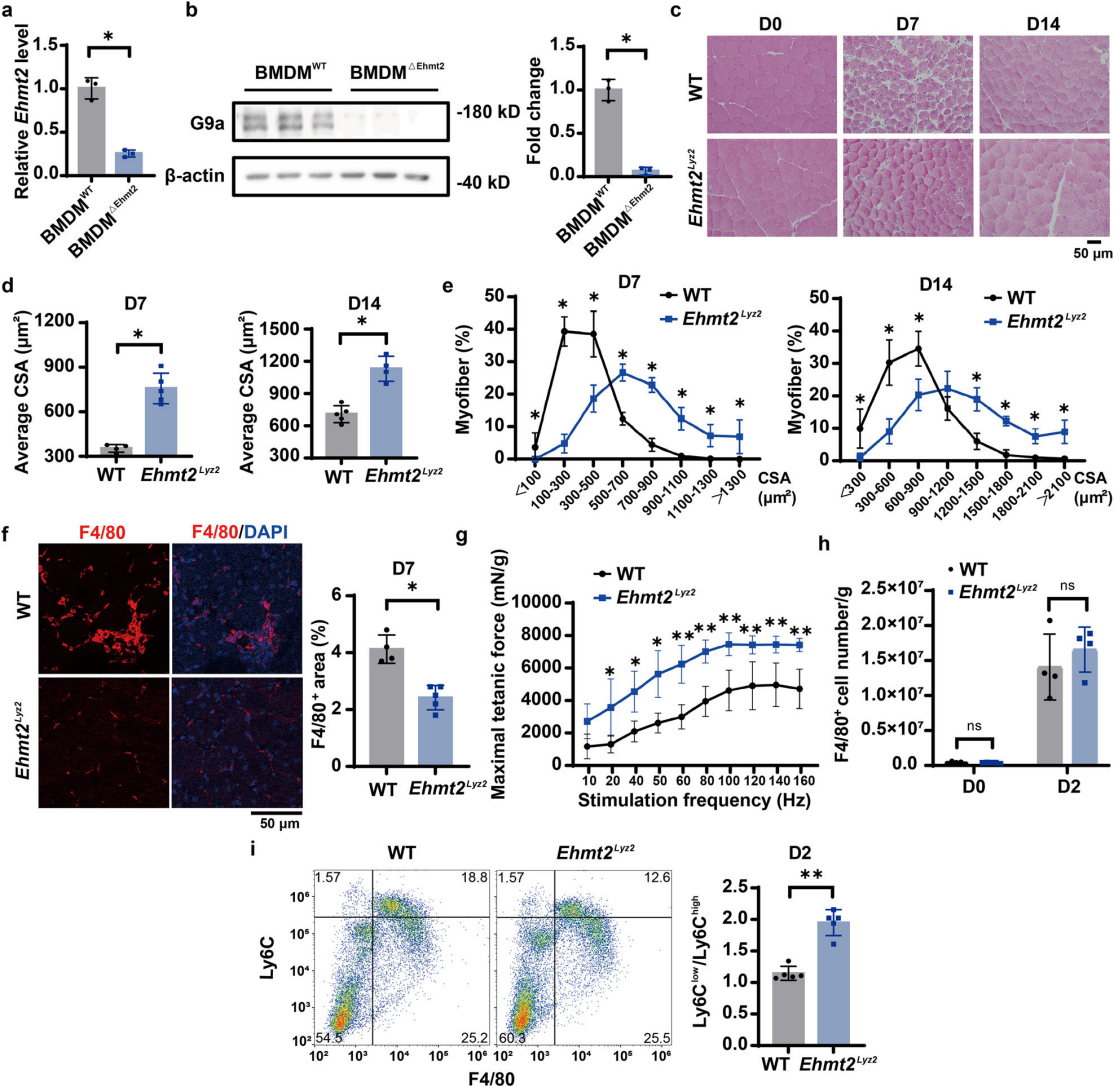
Loss of G9a in myeloid cells accelerates muscle regeneration after CTX-injury. **a** mRNA levels of G9a in the BMDMs isolated from WT or *Ehmt2*^*Lyz2*^ KO mice (BMDMs^WT^ or BMDMs^ΔEhmt2^). **b** Western blots (left) with quantitative analysis (right) of G9a (normalized to β-actin) in the BMDMs^WT^ or BMDMs^ΔEhmt2^. Representative images of H&E staining in the TA muscle of WT and *Ehmt2*^*Lyz2*^ KO mice (**c**), with average myofiber CSA (**d**) and myofiber frequency distribution (**e**) at Day 7 (left) and Day 14 (right) after CTX-injury. **f** Representative F4/80 immunostaining (left) with quantitative analysis (right) in the TA muscle of WT and *Ehmt2*^*Lyz2*^ KO mice at Day 7 after CTX-injury. Red, F4/80 staining; blue, DAPI-stained nuclei. **g** Maximal tetanic force of the TA muscle in WT and *Ehmt2*^*Lyz2*^ KO mice under different frequencies of electrical stimulation at Day 7 after CTX-injury. **h** Number of macrophages (F4/80^+^) per gram (g) of TA muscle in WT and *Ehmt2*^*Lyz2*^ KO mice at Day 0 and Day 2 after CTX-injury. **i** Representative pseudo-color plot from flow cytometry analysis based on F4/80 and Ly6C (left), with the ratio of anti-inflammatory (Ly6C^low^) to pro-inflammatory (Ly6C^high^) macrophages (right) in the TA muscle of WT and *Ehmt2*^*Lyz2*^ KO mice at Day 2 after CTX-injury. BMDMs, bone marrow-derived macrophages; TA tibialis anterior, CTX cardiotoxin, CSA cross-sectional area. *n* = 3-5 per group. Data shown as mean ± SD. The Mann-Whitney test was used for statistical analysis. *P < 0.05, **P < 0.01; ns, no significant difference.

We first investigated the role that myeloid cells G9a plays in muscle regeneration. Myeloid cells-specific G9a knockout (*Ehmt2*^*Lyz2*^ KO) mice were constructed, and significantly reduced transcription and protein levels of G9a were confirmed in bone marrow-derived macrophages (BMDMs) isolated from *Ehmt2*^*Lyz2*^ KO mice (BMDMs^ΔEhmt2^) than those isolated from WT mice (BMDMs^WT^) (Fig. 2a, b). No significant difference in body weight, muscle weight and myofiber morphology was observed between adult *Ehmt2*^*Lyz2*^ KO and WT mice (Fig. S2). After CTX-injury, *Ehmt2*^*Lyz2*^ KO mice showed markedly accelerated muscle regeneration as demonstrated by larger myofiber cross-sectional area (CSA) at Day 7 and Day 14 (Fig. 2c–e), with less infiltrated macrophages at Day 7 (Fig. 2f), as well as increased tetanic forces at Day 7 (Fig. 2g).

Meanwhile, the effects of *Ehmt2*^*Lyz2*^ KO mice in muscle regeneration were also examined in an HLI-injured mouse model. Consistent with the CTX-injury, *Ehmt2*^*Lyz2*^ KO mice also showed larger myofiber CSA, fewer infiltrated macrophages compared to those of WT mice at Day 12 post-HLI (Fig. S3a, b); however, similar vascular density was found in injured muscles from WT and *Ehmt2*^*Lyz2*^ KO mice (Fig. S3c), indicating re-vascularization does not contribute to accelerated muscle regeneration in HLI-injured *Ehmt2*^*Lyz2*^ KO mice.

### Deficiency of G9a in myeloid cells facilitates anti-inflammatory macrophage phenotype transition during muscle regeneration

Because macrophage phenotype transition from pro-inflammatory (M1-like) to anti-inflammatory (M2-like) type is critical for muscle regeneration [9], we next examined whether loss of G9a impacts macrophage phenotype transition after muscle injury. We quantified the total number of macrophages (F4/80^+^), pro-inflammatory (Ly6C^high^) and anti-inflammatory (Ly6C^low^) macrophages in injured muscle at the early inflammatory resolution stage (Day 2 after CTX-injury or Day 4 after HLI-induced injury). Total number of macrophages was comparable between injured muscles of *Ehmt2*^*Lyz2*^ KO and WT mice at the early inflammatory resolution stage (Figs. 2h and S3d), while the ratio of anti-inflammatory to pro-inflammatory macrophages, as indicated by Ly6C^low^/Ly6C^high^, was remarkably boosted in injured muscles of *Ehmt2*^*Lyz2*^ KO mice (Figs. 2i and S3e). These results suggest that ablation of G9a in myeloid cells promoted macrophage phenotype transition, but not macrophage infiltration, during muscle regeneration.

To further validate the effects of G9a on macrophage phenotype, we performed macrophage polarization experiments using BMDMs. Compared to BMDMs^WT^, BMDMs^ΔEhmt2^ exhibited markedly reduced M1-like macrophage markers, as demonstrated by significantly down-regulated transcriptional levels of *iNOS*, *Tnfa* and *IL1b*, accompanied with reduced iNOS protein levels, under LPS plus IFNγ treatment (Fig. S4a, b). Meanwhile, BMDMs^ΔEhmt2^ showed significantly increased M2-like macrophage markers, as demonstrated by significantly up-regulated transcriptional levels of *Arg1* and *Mrc1*, accompanied with increased Arg1 protein levels, under IL4 treatment (Fig. S4c, d). Therefore, in vitro and in vivo results indicate that G9a promotes pro-inflammatory (M1-like) macrophage polarization/transition.

To investigate whether G9a methyltransferase activity dependently regulates macrophage polarization, two structurally different G9a inhibitors, BIX-01294 (BIX) and A366, were used. After exposure to BIX, the levels of H3K9me2, but not G9a and H3K9me1, were significantly decreased in BMDMs (Fig. S5a); and significantly decreased M1-like with increased M2-like macrophage markers were found (Fig. S5b–e). Similar results were obtained after A366 treatment (Fig. S5f–k). Furthermore, overexpressing full-length G9a, but not a SET domain-deficient G9a (G9aΔSET, enzyme-inactivated) (Fig. S5l), up-regulated M1-like and down-regulated M2-like macrophage markers in BMDMs (Fig. S5m–q). These results indicated that G9a regulates macrophage polarization *via* its methyltransferase activity.

### Macrophage G9a affects myotube formation by repressing the expression and secretion of IL13

**Fig. 3:**
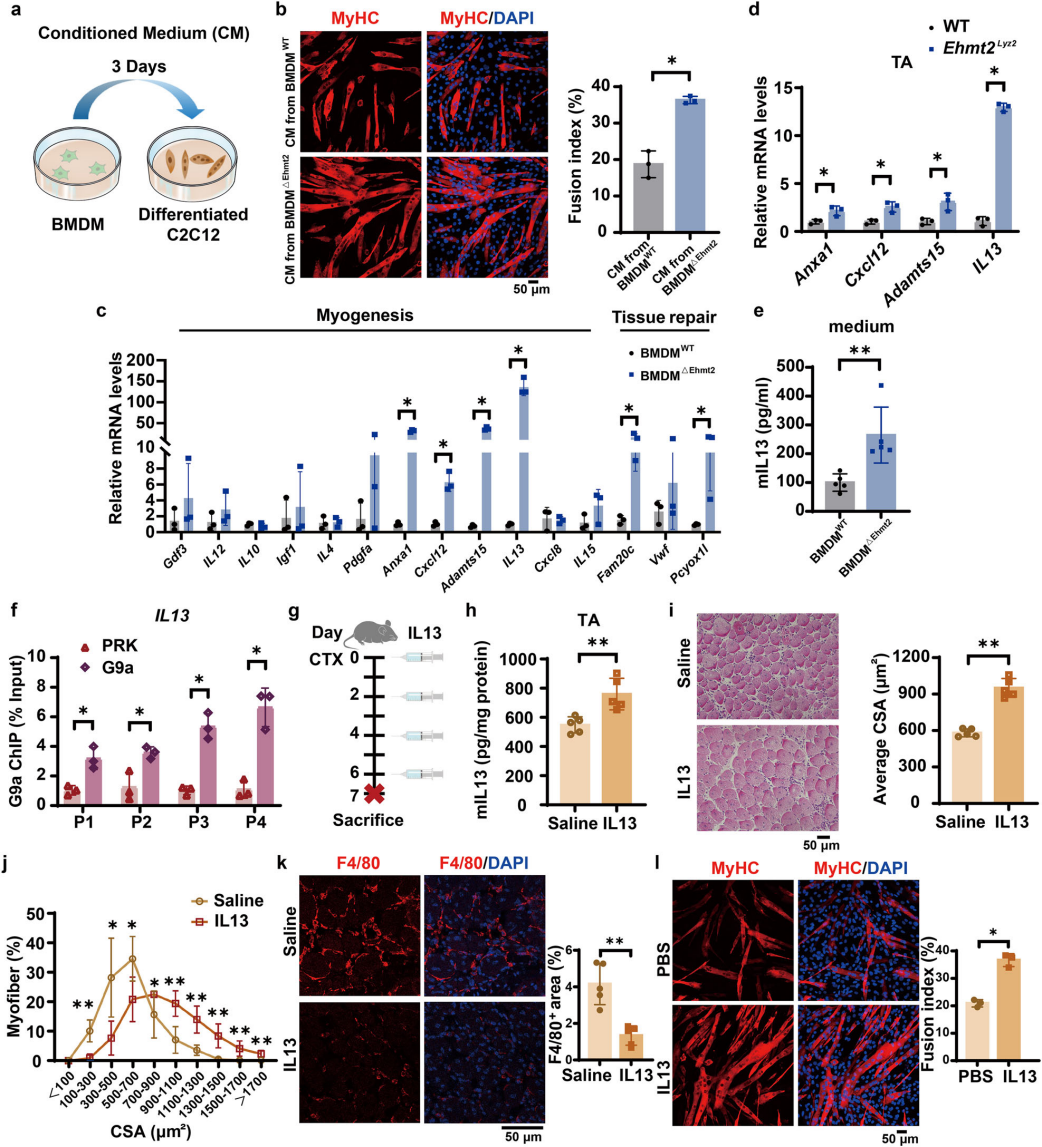
Macrophage G9a represses IL13 level and secretion, which impacts myotube formation. **a** Schematic diagram of co-culture experiments. **b** Representative immunostaining of MyHC (left) and fusion index analysis (right) for C2C12 cells treated with different conditioned medium (CM) from BMDMs which isolated from WT and *Ehmt2*^*Lyz2*^ KO mice (BMDMs^WT^ and BMDMs^ΔEhmt2^). Red, MyHC staining; blue, DAPI stained nuclei. **c** qPCR results of several cytokines in BMDMs^WT^ and BMDMs^ΔEhmt2^. **d** qPCR results of several cytokines in the TA muscle of WT and *Ehmt2*^*Lyz2*^ KO mice. **e** IL13 levels in the medium of BMDMs which isolated from WT and *Ehmt2*^*Lyz2*^ KO mice. **f** G9a binding affinities on the promoter of *IL13* in G9a overexpressed THP-1 cells. **g** Schematic diagram of IL13 treatment experiments. **h** IL13 levels in the TA muscle of C57BL/6 mice treated with or without IL13. Representative images of H&E staining (left, **i**) with average myofiber CSA (right, **i**) and its frequency distribution (**j**), as well as representative F4/80 immunostaining (left, **k**) with quantitative analysis (right, **k**) in the TA muscle of mice treated with or without IL13 at Day 7 after CTX-injury. Red, F4/80 staining; blue, DAPI-stained nuclei. **l** Representative MyHC immunostaining (left) with fusion index (right) in C2C12 cells treated with or without IL13. Red, MyHC staining; blue, DAPI-stained nuclei. BMDMs, bone marrow-derived macrophages; TA tibialis anterior, CSA cross-sectional area. MyHC, Myosin Heavy Chain. *n* = 3-5 per group. Data shown as mean ± SD. Mann-Whitney test was used for statistical analysis. *P < 0.05, **P < 0.01.

Macrophages secrete many cytokines to create an environment favoring muscle regeneration [36], to investigate how macrophage G9a deficiency affects muscle regeneration, co-culture experiments were performed using conditioned medium (CM) collected from BMDMs to treat differentiated C2C12 cells (Fig. 3a). Compared with BMDMs^WT^-derived CM, BMDMs^ΔEhmt2^-derived CM induced more myotube formation in C2C12 cells (Fig. 3b). Several cytokines known involved in myogenesis or tissue repair [9, 11, 36–38] were further investigated. Compared to their respective controls, markedly increased transcriptional levels of *Anxa1* (Annexin A1), *Cxcl12* (C-X-C motif chemokine ligand 12), *Adamts15* (A disintegrin and metalloproteinase with thrombospondin motifs 15) and *IL13* were found in BMDMs and muscles of *Ehmt2*^*Lyz2*^ KO mice at Day 2 after CTX-injury; among which *IL13* was the most dramatically changed (Fig. 3c, d). In the meantime, compared with respective controls, increased IL13 levels were found in the media of BMDMs^ΔEhmt2^ (Fig. 3e). Chromatin immunoprecipitation (ChIP) assays showed that G9a was enriched on the promoter region of *IL13* in THP-1 cells (Fig. 3f). These data suggested that macrophage G9a may be involved in muscle regeneration through inhibiting IL13.

IL13, an important type 2 cytokine mainly secreted by immune cells [39], has been shown to promote myotube formation in vitro [40]. To investigate the effects of IL13 on muscle regeneration in vivo, recombinant mouse IL13 (mIL13) was administered to CTX-injured C57BL/6 mice (Fig. 3g). mIL13 efficiently boosted muscle IL13 levels, and enhanced muscle regeneration capacity as demonstrated by larger myofiber CSA and less macrophage infiltration at Day 7 after CTX-injury (Fig. 3h–k). Consistent with previous reports [40], mIL13 treatment promoted myotube formation in differentiated C2C12 cells (Fig. 3l). All these data indicate that loss of G9a in macrophages accelerates muscle regeneration by enhancing IL13-mediated myogenesis.

### Deficiency of G9a in myeloid cells alters macrophage phenotype transition during muscle regeneration through Stat6

**Fig. 4:**
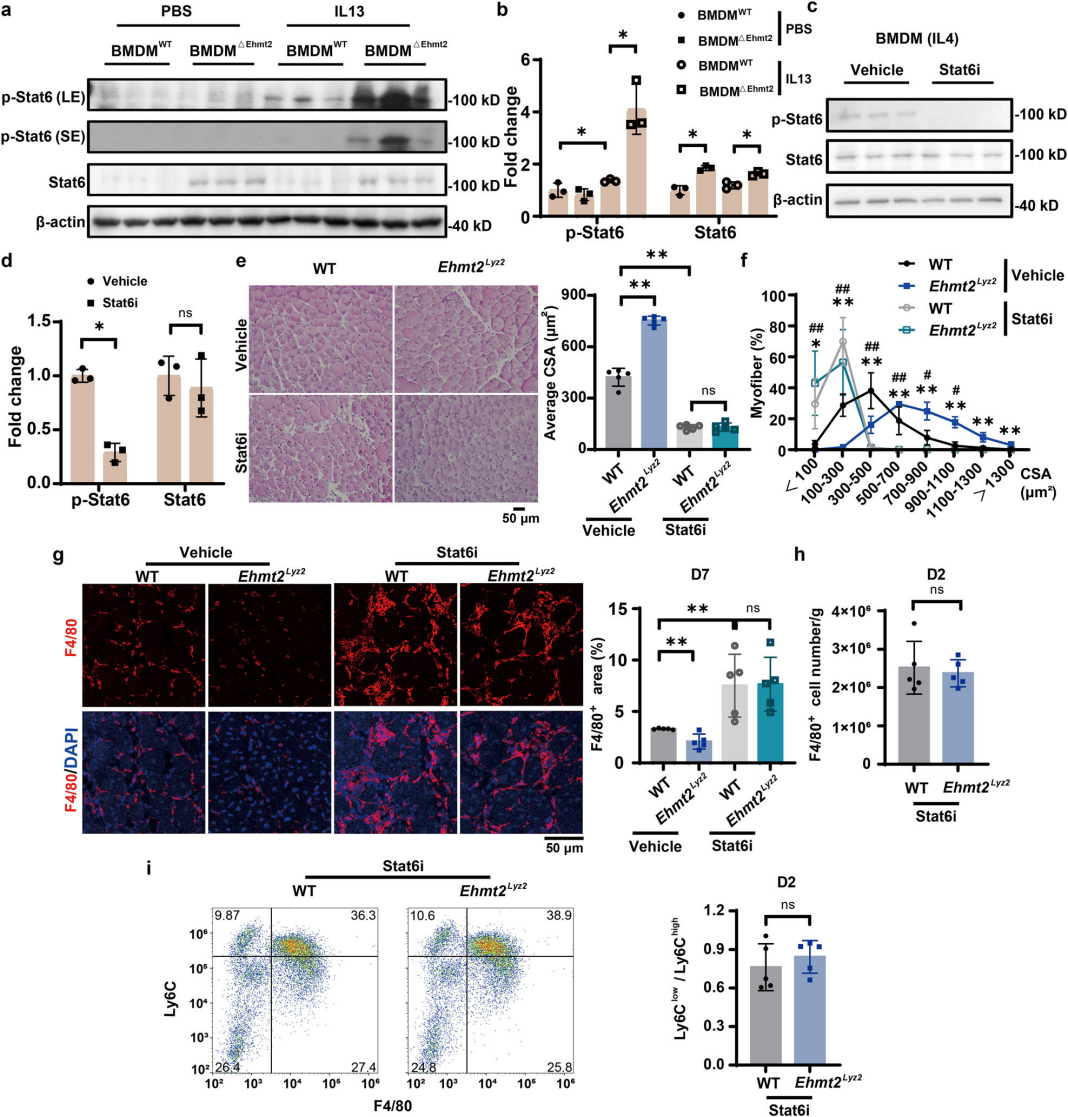
Macrophage G9a alters phenotype transition through Stat6 after CTX-injury. Western blot (**a**) and quantitative analysis (**b**) of Stat6 and p-Stat6 (normalized to β-actin) in the IL13-treated BMDMs isolated from WT or *Ehmt2*^*Lyz2*^ KO mice (BMDMs^WT^ and BMDMs^ΔEhmt2^). Western blot (**c**) and quantitative analysis (**d**) of Stat6 and p-Stat6 (normalized to β-actin) in the BMDMs treated with or without p-Stat6 inhibitor (Stat6i). Representative images of H&E staining (left, **e**) with average myofiber CSA (right, **e**) and its frequency distribution (**f**), as well as F4/80 representative immunostaining (left, **g**) with quantitative analysis (right, **g**) in the TA muscle of WT and *Ehmt2*^*Lyz2*^ KO mice treated with or without Stat6i at Day 7 after CTX-injury. Red, F4/80 staining; blue, DAPI-stained nuclei. **h** Number of macrophages (F4/80^+^) per gram (g) of TA muscle in WT and *Ehmt2*^*Lyz2*^ KO mice treated with or without Stat6i at Day 2 after CTX-injury. **i** Representative pseudo-color plot from flow cytometry analysis based on F4/80 and Ly6C (left) with the ratio of anti-inflammatory (Ly6C^low^) to pro-inflammatory macrophages (Ly6C^high^) (right) in the TA muscle of WT and *Ehmt2*^*Lyz2*^ KO mice treated with or without Stat6i at Day 2 after CTX-injury. LE, long exposure; SE, short exposure; Stat6i, p-Stat6 inhibitor; TA, tibialis anterior; CSA, cross-sectional area; BMDMs, bone marrow-derived macrophages; CTX, cardiotoxin. *n* = 3-5 per group. Data shown as mean ± SD. Mann-Whitney test was used for statistical analysis of two groups, while Kruskal-Wallis test followed by Mann-Whitney test was used for multiple experimental groups. For (**b**–**e,** **g**–**i**): *P < 0.05, **P < 0.01; ns no significant difference. For (**f**): WT+Vehicle *vs. Ehmt2*^*Lyz2*^+Vehicle, *P < 0.05, **P < 0.01; WT+Stat6i *vs*. WT+ Vehicle, ^#^*P* < 0.05, ^##^*P* < 0.01.

Members of Stat family transduce IL13 signaling [39], we thus investigated whether G9a regulates macrophage phenotype transition through this family. Compared to untreated cells, phosphorylated Stat6 (p-Stat6), the active form of Stat6, was significantly increased in both BMDMs^WT^ and BMDMs^ΔEhmt2^ upon IL13 or IL4 treatment, with a further dramatic increase shown in BMDMs^ΔEhmt2^ (Figs. 4a, b and S6a), indicating loss of G9a in macrophage up-regulated Stat6 signaling under type 2 cytokine treatment. Meanwhile, increased Stat6 protein levels were found in injured muscle of WT mice, which were further increased in injured muscle of *Ehmt2*^*Lyz2*^ KO mice at Day 7 after CTX-injury (Fig. S6b). The effect of G9a on Stat6 seems specific, since similar protein levels of the other inflammation-related members of Stat family, such as Stat1 and Stat3, were observed in the injured muscles of WT and *Ehmt2*^*Lyz2*^ KO mice (Fig. S6b).

Next, a p-Stat6 inhibitor (Stat6i) AS1517499 was administrated to CTX-injured mice to investigate whether G9a affects macrophage phenotype transition through Stat6 signaling. Efficiency of AS1517499 was demonstrated by dramatically reduced p-Stat6 in IL4-treated BMDMs isolated from Stat6i-injected mice compared to those from vehicle-injected mice (Fig. 4c, d). AS1517499 treatment abolished the improved muscle regeneration phenotype in *Ehmt2*^*Lyz2*^ KO mice, as demonstrated by similar myofiber CSA and macrophage infiltration between Stat6i-treated WT and *Ehmt2*^*Lyz2*^ KO mice at Day 7 after CTX-injury (Fig. 4e–g). Furthermore, the ratio of anti-inflammatory to pro-inflammatory macrophages (Ly6C^low^/Ly6C^high^), as well as the total number of macrophages, was similar in injured muscles from Stat6i-treated WT and *Ehmt2*^*Lyz2*^ KO mice at Day 2 after CTX-injury (Fig. 4h, i). Meanwhile, similar to myofiber CSA, the number of infiltrated macrophages, and the ratio of anti-inflammatory to pro-inflammatory macrophages (Ly6C^low^/Ly6C^high^) were also found in HLI-injured muscles from Stat6i-treated WT and *Ehmt2*^*Lyz2*^ KO mice (Fig. S7).

Furthermore, compared to those in BMDMs^WT^, the protein and transcriptional levels of *Stat6*, but not *Stat1-3*, were significantly elevated in BMDMs^ΔEhmt2^ (Figs. 4a and S6c). While the transcriptional levels of *IL4ra* and *IL13ra*, the genes encoding receptors for IL4 and IL13 to activate Stat6, were comparable between BMDMs^ΔEhmt2^ and BMDMs^WT^ (Fig. S6d), suggesting G9a may regulate *Stat6* transcription. Consistently, luciferase reporter assays and ChIP assays demonstrated enriched G9a on the promoter region of *STAT6* to regulate its transcription in THP-1 cells (Fig. S6e, f).

### Loss of G9a in myofiber accelerates muscle regeneration and macrophage phenotype transition

**Fig. 5:**
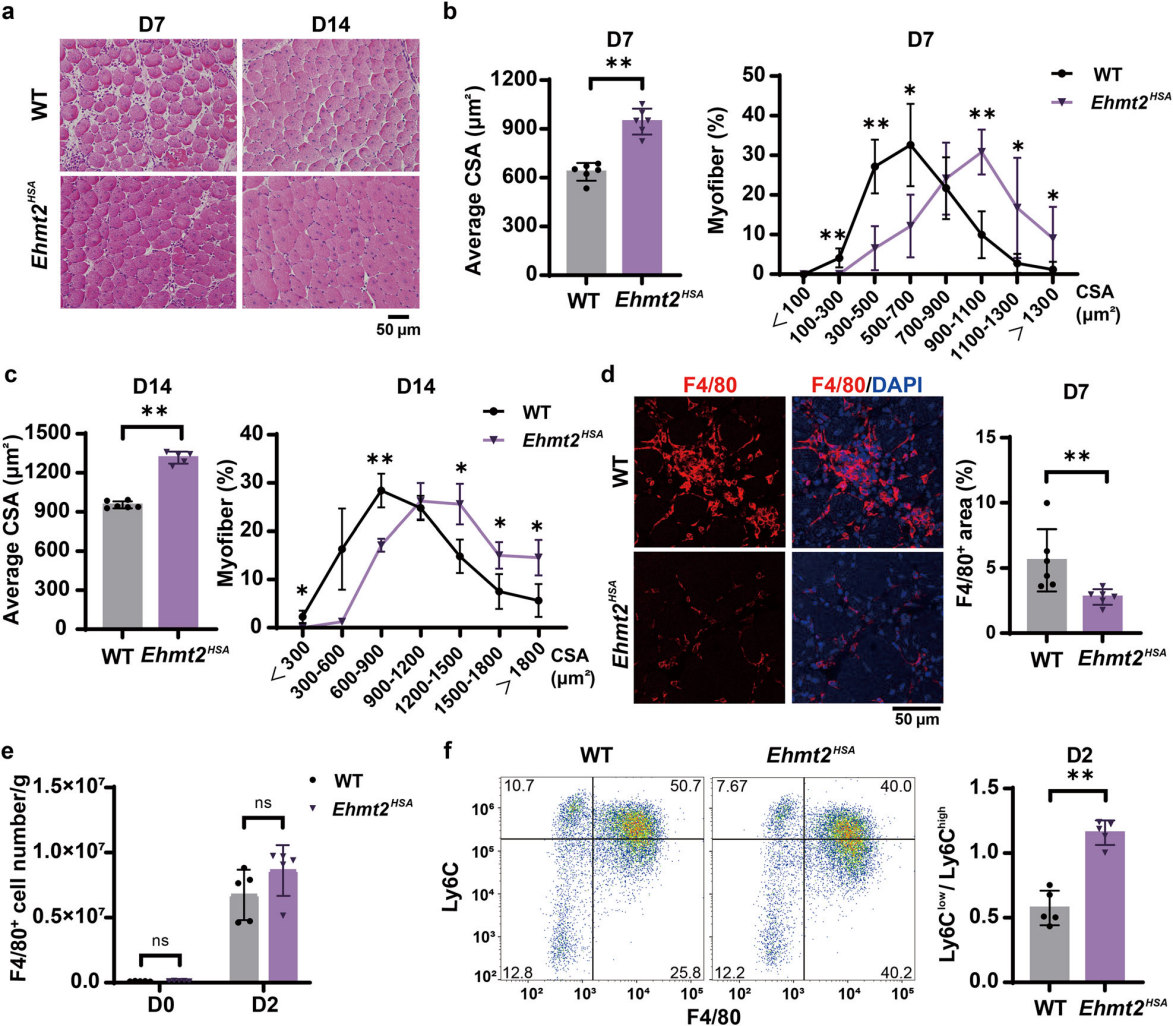
Myofiber G9a deficiency accelerates muscle regeneration and promotes macrophage phenotype transition. **a** Representative images of H&E staining in the TA muscle of WT and *Ehmt2*^*HSA*^ KO mice at Day 7 and Day 14 after CTX-injury. Average myofiber CSA (left) and its frequency distribution (right) in the TA muscle of WT and *Ehmt2*^*HSA*^ KO mice at Day 7 (**b**) and Day 14 (**c**) after CTX-injury. **d** Representative F4/80 immunostaining (left) with quantitative analysis (right) in the TA muscle of WT and *Ehmt2*^*HSA*^ KO mice at Day 7 after CTX-injury. Red, F4/80 staining; blue, DAPI-stained nuclei. **e** Number of macrophages (F4/80^+^) per gram (g) of TA muscle in WT and *Ehmt2*^*HSA*^ KO mice at Day 0 and Day 2 after CTX-injury. **f** Representative pseudo-color plot from flow cytometry analysis based on F4/80 and Ly6C (left), and the ratio of anti-inflammatory (Ly6C^low^) to pro-inflammatory macrophages (Ly6C^high^) (right) in the TA muscle of WT and *Ehmt2*^*HSA*^ KO mice at Day 2 after CTX-injury. TA tibialis anterior, CSA cross-sectional area, CTX cardiotoxin. *n* = 5-6 per group. Data shown as mean ± SD. Mann-Whitney test was used for statistical analysis. *P < 0.05, **P < 0.01; ns, no significant difference.

Because G9a was markedly up-regulated in myofibers after muscle injury (Fig. 1j, k), we next investigated the role of myofiber-expressing G9a in muscle regeneration. Compared with their WT littermates, *Ehmt2*^*HSA*^ KO mice also significantly accelerated muscle regeneration as demonstrated by larger myofiber CSA at Day 7 and 14, with fewer infiltrated macrophages at Day 7 after CTX-injury (Fig. 5a–d). Since the effect of myofibers on macrophage phenotype transition after muscle injury remains largely unknown, we investigated this effect using myofiber-specific G9a-deficient mice. Intriguingly, compared with WT littermates, *Ehmt2*^*HSA*^ KO mice showed a similar total number of macrophages but a significantly increased ratio of anti-inflammatory to pro-inflammatory macrophages (Ly6C^low^/Ly6C^high^) in the muscle at Day 2 after CTX-injury (Fig. 5e, f), which suggests that myofiber G9a suppresses macrophage phenotype transition during muscle injury.

Since aged muscle is accompanied by chronic muscle damage [41], we further examined whether loss of G9a in myofiber protects muscle from aging-related muscle dysfunction. We found down-regulated transcriptional levels of cellular senescence markers *p16* and *p21*, as well as protein levels of p21, in the TA muscle of aged *Ehmt2*^*HSA*^ KO mice (Fig. S8a, b). Moreover, aged *Ehmt2*^*HSA*^ KO mice showed better exercise performance than WT littermates in both running tests, which were performed one day apart (Fig. S8c). Intriguingly, aged *Ehmt2*^*HSA*^ KO mice exhibited superior exercise performance in the second test than the first test, while aged WT mice showed similar capacity in both runs, which indicated loss of G9a may facilitate the repair of aging muscle from the exercise (Fig. S8c).

### Myofiber G9a ablation accelerates muscle regeneration through musclin

**Fig. 6:**
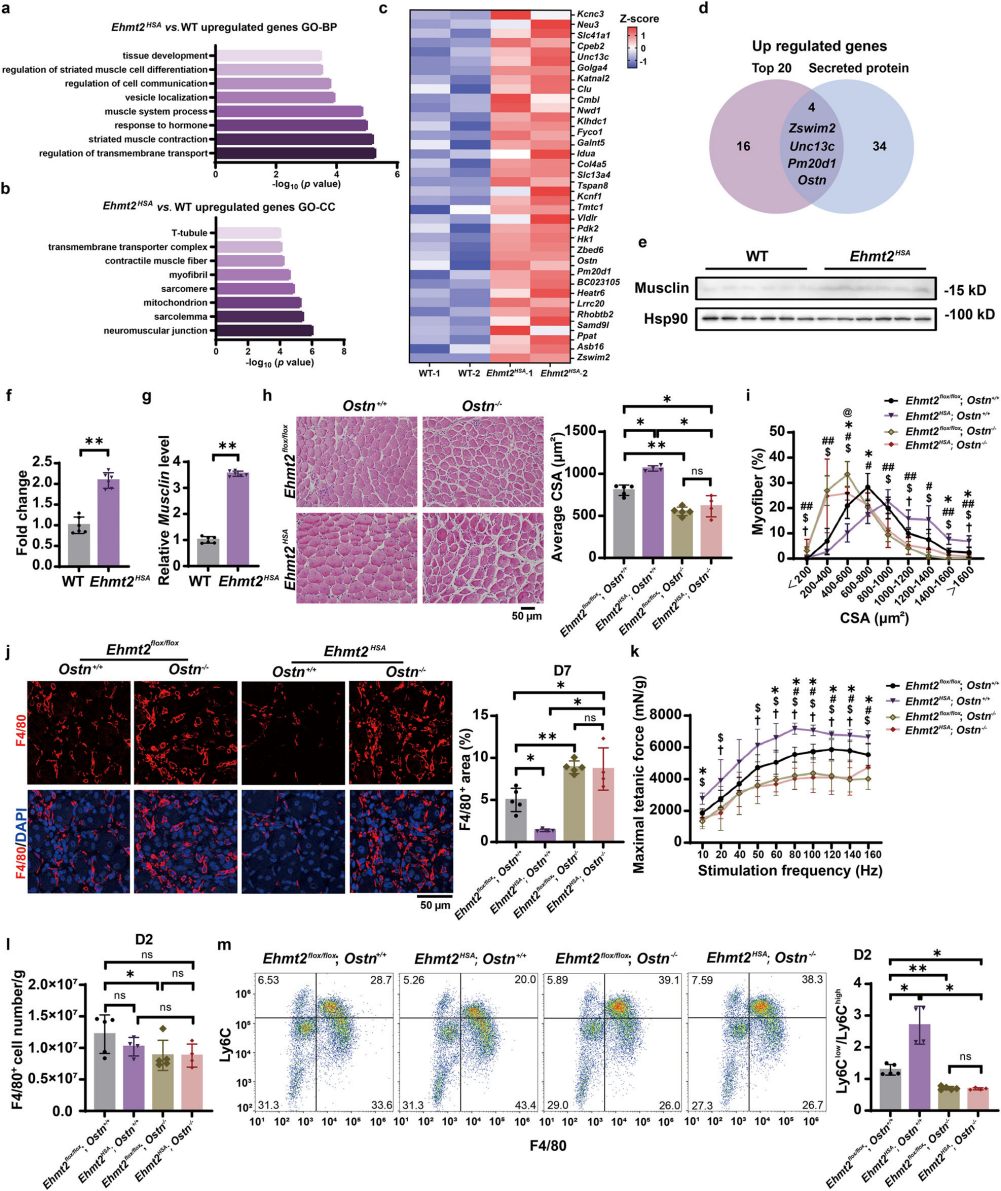
Deletion of Musclin abolishes the improvement effects in *Ehmt2*^*HSA*^ KO mice during muscle regeneration. Gene Ontology (GO) enrichment analysis for biological processes (**a**) and cellular components (**b**) of altered genes in the TA muscle of *Ehmt2*^*HSA*^ KO mice *vs*. WT mice at Day 7 after CTX-injury. **c** Heatmap of up-regulated genes encoding secreted proteins in the TA muscle of WT and *Ehmt2*^*HSA*^ KO mice at Day 7 after CTX- injury. **d** Venn diagrams of the top 20 up-regulated genes and all secreted protein-encoding genes in the TA muscle of *Ehmt2*^*HSA*^ KO mice. Western blot (**e**) and quantitative analysis (**f**) of Musclin (normalized to Hsp90) in the TA muscle of WT and *Ehmt2*^*HSA*^ KO mice at Day 7 after CTX-injury. **g** Transcription levels of *Musclin* in the TA muscle of WT and *Ehmt2*^*HSA*^ KO mice at Day 7 after CTX-injury. Representative images of H&E staining (left, **h**) with average myofiber CSA (right, **h**) and its frequency distribution (**i**), as well as representative F4/80 immunostaining (left, **j**) with quantitative analysis (right, **j**) of indicated groups at Day 7 after CTX-injury. Red, F4/80 staining; blue, DAPI stained nuclei. **k** Maximal tetanic force of the TA muscle in the indicated groups at Day 7 after CTX-injury. **l** Number of macrophages (F4/80^+^) per gram (g) of TA muscle in the indicated groups at Day 2 after CTX-injury. **m** Representative pseudo-color plot from flow cytometry analysis based on F4/80 and Ly6C (left), and the ratio of anti-inflammatory (Ly6C^low^) to pro-inflammatory macrophages (Ly6C^high^) (right) in the TA muscle of the indicated groups at Day 2 after CTX-injury. TA, tibialis anterior; CTX, cardiotoxin; CSA, cross-sectional area. *n* = 4-6 per group. Data shown as mean ± SD. Mann-Whitney test was used for statistical analysis of two groups; while, Kruskal-Wallis test followed by Mann-Whitney test was used for multiple experimental groups. For (**e**–**h,** **j,** **l, m**): *P < 0.05, **P < 0.01; ns, no significant difference; for (**i,** **k**): *Ehmt2*^*flox/flox*^; *Ostn*^+/+^ *vs. Ehmt2*^*HSA*^; *Ostn*^+/+^: *P < 0.05; *Ehmt2*^*flox/flox*^; *Ostn*^+/+^ *vs. Ehmt2*^*flox/flox*^; *Ostn*^-/-^: ^#^*P* < 0.05, ^##^*P* < 0.01; *Ehmt2*^*HSA*;^ *Ostn*^+/+^ *vs. Ehmt2*^*HSA*^; *Ostn*^-/-^: ^$^*P* < 0.05; *Ehmt2*^*flox/flox*^; *Ostn*^+/+^ *vs. Ehmt2*^*HSA*^; *Ostn*^-/-^: ^†^*P* < 0.05; *Ehmt2*^*flox/flox*^; *Ostn*^*-/-*^ *vs*. *Ehmt2*^*HSA*^; *Ostn*^*-/-*^: ^@^*P* < 0.05.

To investigate the mechanism by which myofiber-expressed G9a regulates muscle regeneration, we performed RNA-seq analysis of muscles in the WT and *Ehmt2*^*HSA*^ KO mice at Day 7 after CTX-injury. Gene ontology (GO) analysis for biological process revealed genes up-regulated in *Ehmt2*^*HSA*^ KO mice were mainly enriched in transmembrane transport, cellular communication and muscle tissue development; while GO analysis for cellular components of genes up-regulated in *Ehmt2*^*HSA*^ KO mice also indicated enriched changes in sarcolemma (Fig. 6a, b). Thus, we hypothesized the muscle of *Ehmt2*^*HSA*^ KO mice may secret certain myokines to engage in intercellular crosstalk and accelerate muscle regeneration. Further analysis of RNA-seq data indicated 38 up-regulated genes encoding secreted proteins in *Ehmt2*^*HSA*^ KO mice, four of which were among the top 20 up-regulated genes (Fig. 6c, d). *Ostn* (*Osteocrin*) encodes musclin, a myokine critical for muscle health and regulated by G9a as we previously reported [22], was identified (Fig. 6c, d). Consistently, the transcription and protein levels of musclin were significantly increased in the muscle of *Ehmt2*^*HSA*^ KO mice compared to WT littermates at Day 7 after CTX-injury (Fig. 6e–g).

To determine whether musclin contributes to the improved muscle regeneration in *Ehmt2*^*HSA*^ KO mice, we generated *Ehmt2*^*HSA*^; *Ostn*^-/-^ (DKO) mice in which the musclin-encoding gene *Ostn* is deleted in the *Ehmt2*^*HSA*^ KO background. Success construction of DKO mice was confirmed at the transcriptional and protein levels (Fig. S9a, b). The body weights, muscle weights and myofiber morphology were indistinguishable among adult DKO mice, single KO mice and their controls, indicating musclin is dispensable for muscle development (Fig. S9c–e). However, compared with *Ehmt2*^*HSA*^ KO mice, DKO mice showed robustly smaller myofiber CSA, more infiltrated macrophages and reduced tetanic forces in the muscle at Day 7 after CTX-injury (Fig. 6h–k). A similar total number of macrophages was found among groups (Fig. 6l); however, the improvement in macrophage phenotype transition mediated by myofiber G9a deficiency was abrogated in DKO mice at Day 2 after CTX-injury (Fig. 6m). These results indicated that musclin contributes to the beneficial effects of *Ehmt2*^*HSA*^ KO mice during muscle regeneration.

**Fig. 7:**
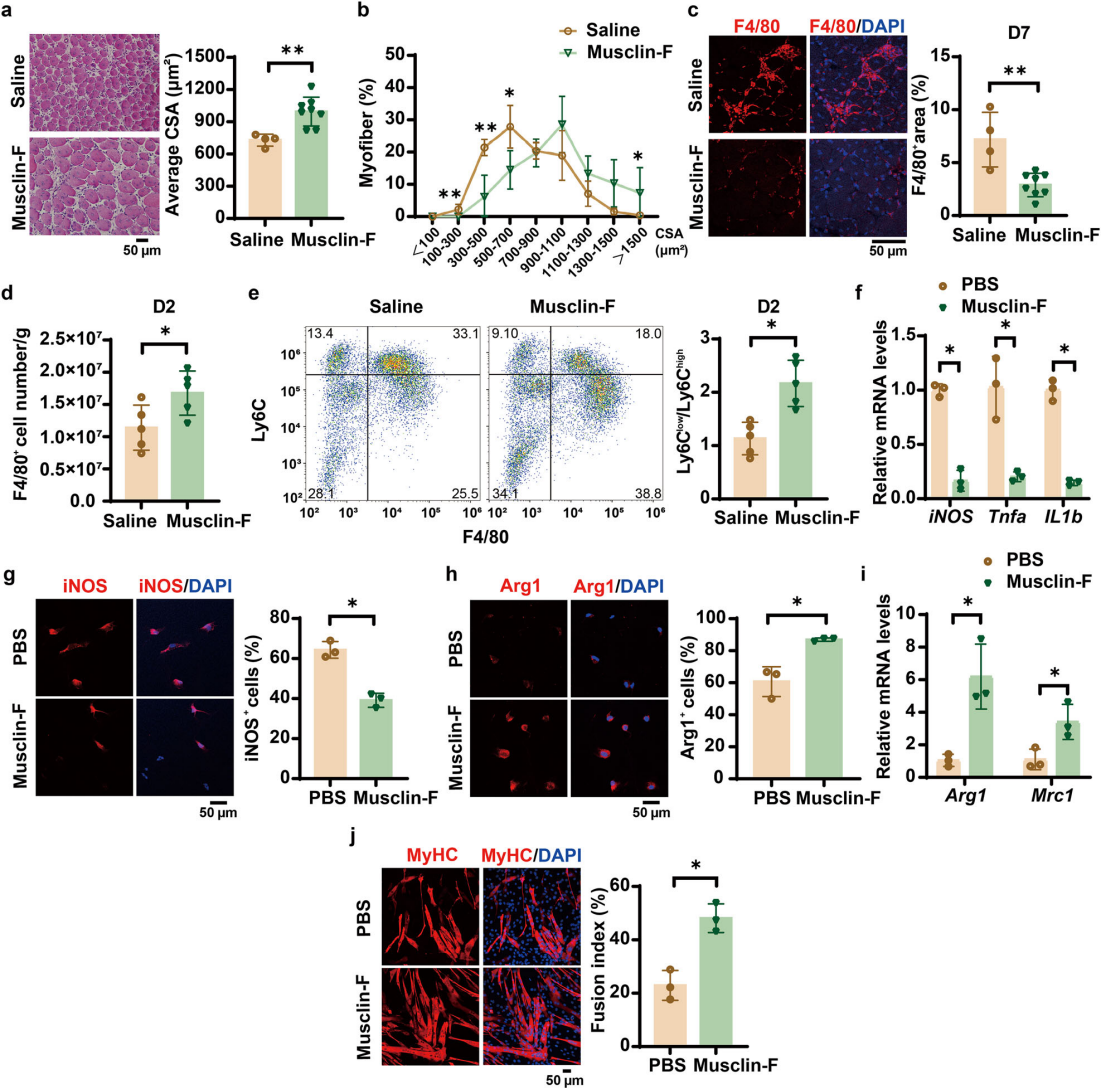
Musclin treatment promotes muscle regeneration. Representative images of H&E staining (left, **a**) with average myofiber CSA (right, **a**) and its frequency distribution (**b**), as well as representative F4/80 immunostaining (left, **c**) with quantitative analysis (right, **c**) in the TA muscle of mice treated with or without Musclin-F at Day 7 after CTX-injury. Red, F4/80 staining; blue, DAPI-stained nuclei. **d** Number of macrophages (F4/80^+^) per gram (g) of TA muscle in mice treated with or without Musclin-F at Day 2 after CTX-injury. **e** Representative pseudo-color plot from flow cytometry analysis based on F4/80 and Ly6C (left), and the ratio of anti-inflammatory (Ly6C^low^) to pro-inflammatory macrophages (Ly6C^high^) (right) in the TA muscle of mice treated with or without Musclin-F at Day 2 after CTX-injury. Transcriptional levels of M1-like macrophage markers (**f**) and representative iNOS immunostaining (left, **g**) with quantitative analysis (right, **g**) in BMDMs treated with or without Musclin-F. Red, iNOS staining; blue, DAPI-stained nuclei. Representative Arg1 immunostaining (left, **h**) with quantitative analysis (right, **h**) and transcriptional levels of M2-like macrophage markers (**i**) in BMDMs treated with or without Musclin-F. Red, Arg1 staining; blue, DAPI-stained nuclei. **j** Representative MyHC immunostaining (left) and fusion index (right) in C2C12 cells treated with or without Musclin-F. Red, MyHC staining; blue, DAPI-stained nuclei. TA, tibialis anterior; CSA, cross-sectional area; BMDMs, bone marrow-derived macrophages; CTX, cardiotoxin; MyHC, Myosin Heavy Chain. *n* = 3-8 per group. Data shown as mean ± SD. Mann-Whitney test was used for statistical analysis. *P < 0.05, **P < 0.01.

To investigate the role that musclin plays in muscle regeneration and its potential as a therapeutic treatment for related disorders, full-length musclin (Musclin-F) or its core peptide (Musclin-33) [42] was administrated to C57BL/6 mice under CTX-injury. Compared to untreated mice, Musclin-F effectively improved the myofiber CSA and reduced macrophage infiltration in the muscle at Day 7 after CTX-injury (Fig. 7a–c). Furthermore, increased number of macrophages and increased ratio of anti-inflammatory to pro-inflammatory macrophages (Ly6C^low^/Ly6C^high^) were found in Musclin-F-treated mice at Day 2 after CTX-injury (Fig. 7d, e). In vitro studies also demonstrated decreased M1-like markers and increased M2-like macrophage markers in BMDMs treated with Musclin-F (Fig. 7f–i). Moreover, significantly promoted myotube formation was also found in C2C12 cells treated with Musclin-F (Fig. 7j). Similar effects were found after Musclin-33 treatment (Fig. S10).

### IL13 and Musclin combination synergistically enhances muscle regeneration

**Fig. 8:**
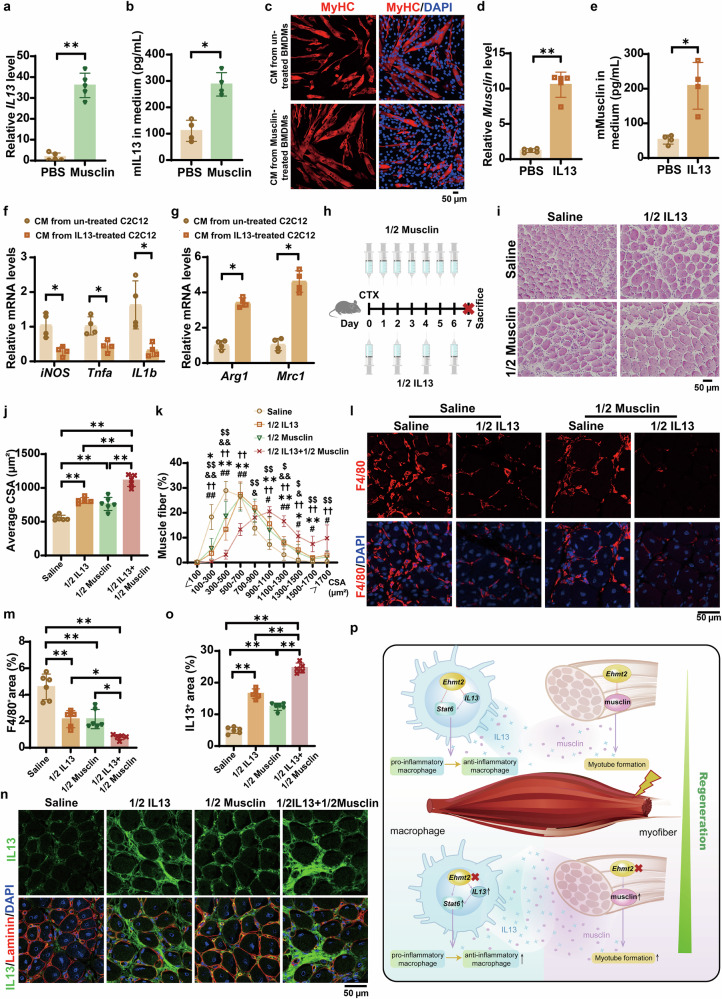
Combined administration of IL13 and Musclin show better effects on accelerating muscle regeneration. **a** Transcription levels of *IL13* in the BMDMs treated with or without Musclin-F. **b** IL13 levels in the medium of BMDMs treated with or without Musclin. **c** Representative MyHC immunostaining in C2C12 cells treated with conditioned medium from musclin‑treated or untreated BMDMs (CM from musclin‑treated or untreated BMDMs). Red, MyHC staining; blue, DAPI-stained nuclei. **d** Transcription levels of *Musclin* in the C2C12 cells treated with or without IL13. **e** Musclin levels in the medium of C2C12 cells treated with or without IL13. Transcriptional levels of M1-like macrophage markers (**f**) and M2-like macrophage markers (**g**) in BMDMs treated with conditioned medium from IL13‑treated or untreated C2C12 cells (CM from IL13‑treated or untreated C2C12). **h** Schematic diagram for combined IL13 and Musclin administration in mice. Representative images of H&E staining (**i**) with average myofiber CSA (**j**) and its frequency distribution (**k**) in the TA muscle of the indicated groups at Day 7 after CTX-injury. Representative F4/80 immunostaining (**l**) with quantitative analysis (**m**) in the TA muscle of the indicated groups at Day 7 after CTX-injury. Red, F4/80 staining; blue, DAPI-stained nuclei. Representative IL13 immunostaining (**n**) with quantitative analysis (**o**) in the TA muscle of the indicated groups at Day 7 after CTX-injury. Green, IL13 staining; red, Laminin staining; blue, DAPI-stained nuclei. **p** A schematic model of how G9a regulates muscle regeneration. TA, tibialis anterior; CSA, cross-sectional area. *n* = 5–6 per group. Data shown as mean ± SD. Mann-Whitney test was used for statistical analysis of two groups; while, Kruskal-Wallis test followed by Mann-Whitney test was used for multiple experimental groups. For (**a**–**j, m**–**o**): *P < 0.05, **P < 0.01; for **k**: Saline *vs*. 1/2 IL13, ^$^*P* < 0.05, ^$$^*P* < 0.01; Saline *vs*. 1/2 Musclin, ^&^*P* < 0.05, ^& &^*P* < 0.01; Saline *vs*. 1/2 IL13 + 1^/^2 Musclin, ^††^*P* < 0.01; 1/2 IL13 *vs*. 1/2 IL13 + 1/2 Musclin, *P < 0.05, **P < 0.01; 1/2 Musclin *vs*. 1/2 IL13 + 1/2 Musclin, ^#^*P* < 0.01, ^##^*P* < 0.01.

Notably, dramatically increased transcription and protein levels of IL13 were found in cell lysate and medium of BMDMs treated with Musclin-F (Fig. 8a, b). Furthermore, conditioned medium collected from Musclin-F-treated BMDMs (CM from Musclin-treated BMDMs) significantly promoted myotube formation in C2C12 cells compared to that of CM from untreated BMDMs (Figs. 8c and S11a), which indicated factors such as IL13 secreted by BMDMs contribute to myotube formation. Meantime, markedly increased transcription and protein levels of Musclin were also found in mIL13-treated C2C12 cell lysate and medium (Fig. 8d, e). Furthermore, conditioned medium from IL13‑treated C2C12 cells (CM from IL13‑treated C2C12) markedly suppressed M1-like macrophage polarization while enhancing M2-like macrophage polarization compared to that of CM from untreated C2C12 cells (Figs. 8f, g and S11b–e), suggesting factors secreted by C2C12 cells, such as musclin, regulate macrophage polarization. All these results implicate a macrophage-myofiber interplay mediated by these secreted factors during muscle regeneration, at least in vitro.

Furthermore, we investigated whether IL13 and musclin show synergistic beneficial effects. As shown above, administration of half dosage of IL13 or Musclin-F also improved muscle regeneration as demonstrated by increased myofiber CSA (Fig. 8h–k) and decreased macrophage infiltration (Fig. 8l–m) at Day 7 after CTX-injury; while combined administration of IL13 and Musclin-F synergistically improved muscle regeneration more than either one used alone, as indicated by the modified probability sum test (Fig. 8i–k). Furthermore, immunofluorescent staining showed that the level of IL13, which was low in injured muscle, was significantly increased after Musclin- or IL13-treatment, and was further increased after combined treatment of Musclin and IL13 at Day 7 after CTX-injury (Fig. 8n, o). Moreover, increased IL13 was mainly located at inter-myofibers with some were co-stained with the membrane of the myofibers, indicating IL13 may bind to certain receptor(s) on the myofiber membrane to initiate signaling transduction (Fig. 8n, o).

## Discussion

Its extraordinary regenerative capacity makes skeletal muscle an excellent paradigm for tissue repair, whereas this capacity is compromised in muscle degenerative disorders. Defects in muscle regeneration generally cause incomplete regeneration in muscle morphology and functions, even some reparative disorders, including adipocyte accumulation, fibrosis and heterotopic ossification [43]. Thus, understanding key factors in muscle regeneration enables a better cure. Herein, using myeloid cells-specific G9a knockout and myofiber-specific G9a knockout mice, we showed G9a as a critical negative regulator of muscle regeneration by regulating the regeneration microenvironment *via* mediating crosstalk between myofibers and macrophages (Fig. 8p).

Muscle regeneration microenvironment depends on proper crosstalk among multiple cell types within injured muscle [7, 44]; its disruption may impair muscle regeneration [43]. During muscle regeneration, endothelial cells act on satellite cells and myoblasts by secreting angiopoietin-1 (Ang-1), IGF-1, platelet-derived growth factor (PDGF), and vascular endothelial growth factor (VEGF), thereby promoting myogenesis [44]. In addition, myofibers, FAPs, Treg cells, and fibroblasts also secrete cytokines to regulate myogenesis [13, 44]. Furthermore, macrophages act on multiple cell types, including MuSCs, myoblasts, and FAPs by secreting abundant cytokines such as IL6, IL10, IGF-1, GDF3 (growth differentiation factor 3), and oncostatin M [45]. Meanwhile, phagocytosis of necrotic myofibers by macrophages drives their phenotypic switch toward an anti-inflammatory type during muscle regeneration [9, 46]. However, the crosstalk between macrophages and myofibers remains largely undefined during this process. Here, we found a novel interplay between macrophage-secreted IL13 and myofiber-secreted Musclin, both regulated by G9a, that provides a favorable microenvironment for muscle regeneration (Figs. 3, 6 and 8).

G9a has been shown to play a vital role in maintaining the integrity of muscle morphology and function. Previously, we reported loss of G9a in myofibers prevents obesity and hepatic steatosis through epigenetic regulation of Musclin [22], as well as ameliorates aging-associated or denervation-induced muscular atrophy by maintaining the integrity of the neuromuscular junction [24]. While global inhibition of G9a methyltransferase activity by A366 promotes muscle regeneration [19], G9a deficiency in MuSCs or myoblasts show no effect [21], letting us identifying critical muscular cell types that regulate muscle regeneration in the absence of G9a. By analyzing single-cell RNA-seq and Western blots, we found dynamic alterations of G9a in different muscular cells during muscle regeneration process (Figs. 1 and S1). Furthermore, we demonstrated G9a ablation in macrophages or myofibers accelerated muscle regeneration in vivo and in vitro (Figs. 2, 3, 5 and S3). Herein, we clarified two cell-type-specific roles of G9a during muscle regeneration.

IL13 is a cytokine secreted by immune cells that serves multiple protective effects, such as enhancing smooth muscle contractility, inhibiting macrophage immunosenescence and improving angiogenesis [47–50]. IL13 has been shown to promote cardiac repair after myocardial infarction [51]. Here, we demonstrated that IL13 accelerated muscle repair through promoting myogenesis (Fig. 3). Stat6 transduces IL13 signaling [39] and has been shown to involve in tissue repair by enhancing optic nerve regeneration, epithelial and mucosal repair [52–54]. Herein, inhibition of Stat6 facilitated macrophage phenotype transition and muscle regeneration, which suggests that IL13-Stat6 axis mediated anti-inflammatory effect in macrophages is crucial for muscle repair (Figs. 4 and S7). However, how macrophage-secreted IL13 affects myofibers during muscle regeneration remains unclear. Whether its receptors IL13RA1 and IL4RA play roles in myogenesis awaits further investigation.

Myokines, secreted by muscle, have been reported to regulate myogenesis, osteogenesis, and metabolism in autocrine, paracrine, and endocrine ways; however, their effects in regulating muscle regeneration are rarely studied [55]. Musclin, a myokine secreted by adult muscle and neonatal bone, shows multiple beneficial effects on health, including improving physical endurance, preventing obesity and hepatic steatosis, as well as alleviating muscle loss in cancer cachexia [22, 42, 56]. Moreover, it prevents fatty and fibrotic tissue accumulation in muscles after exercise by weakening proliferation and promoting apoptosis for FAPs [57]. Here, we identified a novel beneficial effect of musclin on muscle regeneration by affecting myogenesis on myofibers and promoting anti-inflammatory phenotype transition of macrophages (Fig. 7). Musclin is a ligand for natriuretic peptide receptor C (NPR-C), which increases atrial natriuretic peptide (ANP) level and inhibits the secretion of inflammatory mediators in macrophages [58]. Thus, we hypothesized that during muscle regeneration, myofiber-derived Musclin may act on macrophage NPR-C to increase ANP, thereby modulating macrophage phenotype transition. However, further experiments are needed to verify this hypothesis.

Taken together, we revealed a novel mechanism of muscle regeneration in which G9a inhibits muscle regeneration through disturbing IL13/musclin-mediated intercellular communications, which provide potential therapeutic interventions for muscle regeneration defects.

## Supplementary information

Supplementary data

Full and uncropped Western blots

## Data Availability

All data are available in the main text or the supplementary materials. RNA-seq data of this study are available in the NCBI GEO database (accession code GSE299523).
