# Supplementary material for: Deficiency of G9a boosts muscle regeneration through IL13/Musclin-mediated crosstalk between macrophage and myofiber
Source: Cell Death Dis. 2026 Jun 10;17(1):728. doi: 10.1038/s41419-026-08944-2 (PMC13473583; doi:10.1038/s41419-026-08944-2)
Supplement: Supplementary file 1 [file 41419_2026_8944_MOESM1_ESM.pdf]

Supplemental Materials

for

Deficiency of G9a boosts muscle regeneration through IL13/Musclin-mediated crosstalk between macrophage and myofiber

Ying Jin[1], Kaiyang Zhou[1], Siyu Hao[2], Hu Yue[2], Haoyu Wang[3], Zhiyuan Liu[1], Yihao Zhou[1], Yunhao Xie[1], Xinran Liu[2], Hong Chen[2], Yuxia Liu[1], Anlin Peng[4], Yangkai Li[5], Kun Huang[2]*, Ling Zheng[1]*

[1]State Key Laboratory of Metabolism and Regulation in Complex Organisms, TaiKang Center for Life and Medical Sciences; Frontier Science Center for Immunology and Metabolism, College of Life Sciences, Wuhan University, Wuhan, China, 430072

[2]School of Pharmacy, Tongji Medical College, Huazhong University of Science and Technology, Wuhan, China, 430030

[3]Division of Life Science, State Key Laboratory of Molecular Neuroscience, The Hong Kong University of Science and Technology, Hong Kong SAR, China, 999077

[4]Wuhan Third hospital, Tongren Hospital of Wuhan University, Wuhan, China, 430060

[5]Department of Thoracic Surgery, Tongji Hospital, Tongji Medical College, Huazhong University of Science and Technology, Wuhan, China, 430030

Corresponding Authors

Kun Huang, Ph.D., Tongji School of Pharmacy, Huazhong University of Science & Technology, Wuhan, China, 430030, kunhuang@hust.edu.cn

Ling Zheng, Ph.D., College of Life Sciences, Wuhan University, Wuhan, China 430072,
lzheng@whu.edu.cn

This Supplemental Materials file contains 2 supplemental tables and 11 supplemental figures.

Table S1 Primers used in this study

Table S2 Antibodies used in this study

Figure S1 G9a is dynamically altered after HLI-induced muscle injury.

Figure S2 Similar body weights, muscle weights and myofiber morphology between young WT and *Ehmt2*^{Lyz2} KO mice.

Figure S3 Loss of G9a in myeloid cells accelerates muscle regeneration after HLI-induced muscle injury.

Figure S4 Loss of G9a in myeloid cells affects macrophage polarization.

Figure S5 G9a regulates macrophage polarization in a methyltransferase activity-dependent manner.

Figure S6 G9a binds to the *STAT6* promoter and inhibits its transcription.

Figure S7 Macrophage G9a inhibits the transition to an anti-inflammatory phenotype *via* Stat6 after HLI-induced muscle injury.

Figure S8 *Ehmt2*^{HSA} KO mice ameliorate aging-induced muscle functional loss.

Figure S9 Similar body weights, muscle weights and myofiber morphology between *Ehmt2*^{HSA}; *Ostn*$^{-/-}$ and *Ehmt2*^{HSA}; *Ostn*$^{+/+}$ mice.

Figure S10 Musclin-33 accelerates muscle regeneration.

Figure S11 IL13 and Musclin mediate crosstalk between macrophages and myofibers *in*

vitro.

Table S1. Primers used in this study

Gene	Forward	Reverse
**Primers for qPCR**		
M *Ehmt2*	GGTGAAGCCATCTAGAAAACGG	GAACCTCCATGTACTCACTGG
M *Stat6*	GAGCTACTGGTCAGATCGGC	GGATGACGTGTGCAATGGTG
M *Anxa1*	GCCGCGTACTTACAGGAGAA	ATGGCACCACGGAGTTCATC
M *IL13*	GGCAGCATGGTATGGAGTGT	CTTGCGGTTACAGAGGCCAT
M *Adamts15*	CGTATTGCCTTGGGCTCTCA	AGCAGGGTCAGGGTGAATTG
M *Cxcl8*	CTCCAAACCTTTCCACCCCA	CTCCAAACCTTTCCACCCCA
M *Gdf3*	GATGAGTGTGGGTGTGGGTAG	CACCAAGGGGTCCATAGTCAT
M *IL15*	CAGGTCCTCCTGCAAGTCTC	TTTCCTGACCTCTCTGAGCTG
M *IL12*	GGTTCTTCGAGAGCCACATC	TCTTCAGCCGTGCAACAA
M *IL10*	GCTATGCTGCCTGCTCTTACT	CCTGCTGATCCTCATGCCA
M *Fam20c*	GAATCCTGACTGGCCGCAT	TGCCAATGTGGAATTTGAGCC
M *Vwf*	TGGGTTTTCTCTCCCTGGCT	GCCCATACAAACAGGGGCT
M *Pdgfa*	GGTACTGAATTTCGCCGCCA	GGGTATCTCGGCTTCCTCG
M *Pcyox1l*	CCTCCTTTCACTCTCTGAGCCT	GCGGAAGAGATTCAGCAGGTAC
M *Igf1*	AGAGCCTGCGCAATGGAATA	TGCTGATTTTCCCCATCGCT
M *Musclin*	CCCCTTGACAGACTCTCAGC	GCCGGTTTCTACCAATCCGA
M *IL4*	CCAAACGTCCTCACAGCAAC	AGGCATCGAAAAGCCCGAA
M *Cxcl12*	GGTGCTCAAACCTGACGGTA	GGCAGCTCCTCTTTGGCTTA
M *IL4ra*	ACGTGGTACAACCACTTCCA	GAACAGGCAAAACAACGGGA
M *Rn18s*	CACCATCATGCAGAACCCACGAC	AGCCTCTCCAGGTCCTCACGC
M *IL13ra1*	CAGGTGGGCTCTCAGTGTAG	GCGGACTCAGGATCACCTTC
M *Stat1*	CTGTCATCCCGCAGAGAGAA	TGGAGCAGAGCTGAAACGAC
M *Stat2*	GGTGGAAAACGGGTCCTTGA	TGCGCCATTTGGACTCTTCT
M *Stat3*	TGTGTGACACCATTCATTGATGC	ACACTCCGAGGTCAGATCCA
M *p16*	CGAACTCGAGGAGAGCCATC	GGGGTACGACCGAAAGAGTT
M *p21*	TGAATGGAGACAGAGACCCCA	GGTCGGACATCACCAGGATT

**Primers for ChIP**		
H-*STAT6*-P1	GAGGGAGGAGGGGAGTAGAA	ACTCCCGGACACCTGAGAG
H-*STAT6*-P2	ACTCCCGGACACCTGAGAG	AGCTGGTCTTTGCTCTCTCC
H-*STAT6*-P3	CGCAGAGTCCAGAGGGATTT	ACTCTGTCCCCCTCAAAAGC
H-*STAT6*-P4	GAACTCGCTGGACAGAGCTA	CATCTCTTGGGGACCATCCA
H-*IL13*-P1	CGTGAAGACAGTGACGCTG	TGGCTCACGCCTGTAATCC
H-*IL13*-P2	AATGCCAGAGGCCCATAGAG	TTGGCAAAGACATGATGCCC
H-*IL13*-P3	TTGGCAAAGACATGATGCCC	CATGTCAGGCCGAGGGACC
H-*IL13*-P4	CGTGGGCCCTCTACTACAGAT	TTCCCACAGGGAACGTCATT
**Primers for genotyping**		
Ehmt2-flox	TGTGAGTTCCAGGTAGTGGC	GAATGCCACACAGCAGTGAC
Lyz2-Cre	CCCAGAAATGCCAGATTACG	GGAAGATCAAGTGCTGAAGTCC
	TTACAGTCGGCCAGGCTGAC	GGAAGATCAAGTGCTGAAGTCC
HSA-Cre	GAACCTGATGGACATGTTCAGG	AGTGCGTTCGAACGCTAGAGCCT
Ostn-1	CTCAGACTTTTATGCCTAGCATGAAG	CAAGTGTGCTTCAAAACAAATTCTG
Ostn-2	AACAGGCATGTCATCTAGTGGTAGG	ATGTGGGATCATGTTGCAGACTC

Table S2. Antibodies used in this study

Antibody	Company	Catalog number
G9a (WB/ChIP)	Abcam	185050
H3K9me2 (WB)	Abcam	1220
H3K9me1 (WB)	Abcam	9045
H3 (WB)	ABclonal	A2348
Laminin (IF)	Sigma	L9393
F4/80 (IF)	Cell Signaling Technology	70076
CD31 (IF)	Cell Signaling Technology	77699
iNOS (IF)	BD Pharmingen	BD610431
Arg1 (IF)	Santa Cruz	sc271430
MyHC (IF)	Santa Cruz	sc376157
p21 (IF)	Cell Signaling Technology	2947
IL13 (IF)	Invitrogen	PA5-96053
Stat6 (WB)	Cell Signaling Technology	9032
p-Stat6 (WB)	Cell Signaling Technology	9031
Stat3 (WB)	Cell Signaling Technology	4904
p-Stat3 (WB)	Cell Signaling Technology	9145
Stat1 (WB)	Cell Signaling Technology	9172
p-Stat1 (WB)	Cell Signaling Technology	7649
Musclin (WB)	R&D systems	MAB2620
β-actin (WB)	Proteintech	66079
Hsp90 (WB)	Santa Cruz	13119
α-Tubulin (WB)	Beyotime	AF0001
Rabbit IgG (WB)	Bio-Rad	1706515
Mouse IgG (WB)	Bio-Rad	1706516
Rat IgG (WB)	Bio-Rad	HAF005
Rabbit Alexa 594 IgG (IF)	Invitrogen	A11012
Rabbit Alexa 488 IgG (IF)	Invitrogen	A11008

Mouse Alexa 594 IgG (IF)	Invitrogen	A11005
CD16/CD32 (Flow)	BD Pharmingen	553141
PE F480 (Flow)	BD Pharmingen	565410
APC Ly6C (Flow)	BD Pharmingen	560595

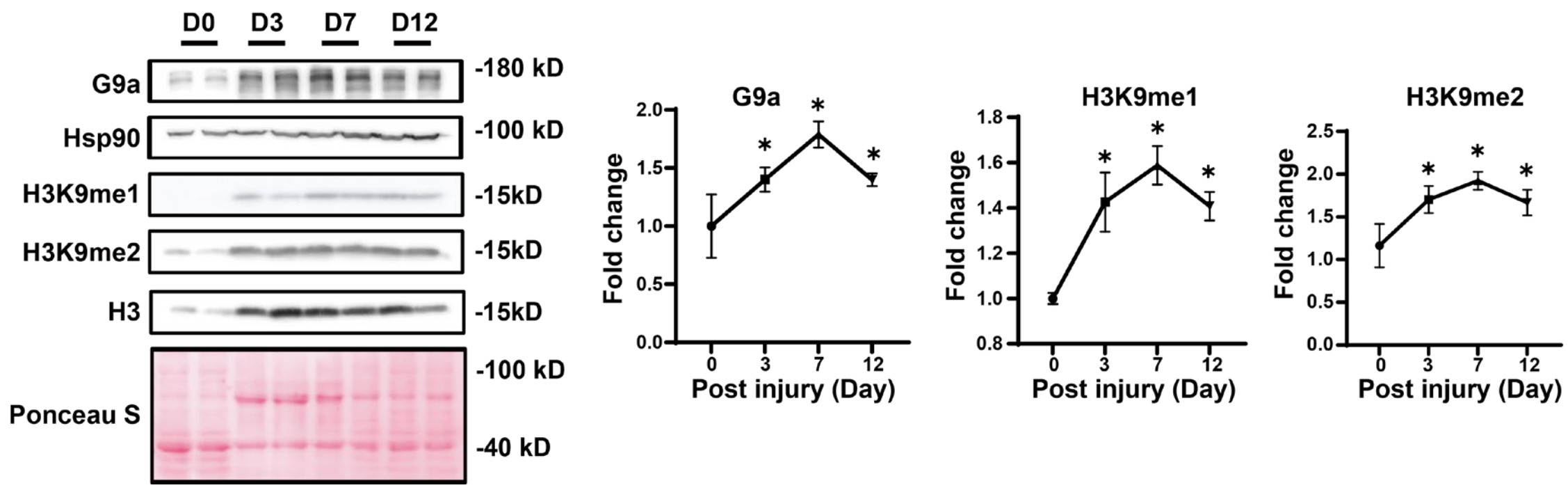

Figure S1 G9a is dynamically altered after HLI-induced muscle injury. Western blots (left) with quantitative analysis (right) of G9a (normalized to Hsp90) and H3K9me1/2 (normalized to histone H3) in the TA muscle of C57BL/6 mice at indicated times post-HLI. Ponceau S staining was used as an additional loading control to confirm equal protein loading. TA, tibialis anterior; HLI, hindlimb ischemia. n = 4 per group. Data shown as mean ± SD. Mann-Whitney test was used for statistical analysis. *$P < 0.05$.

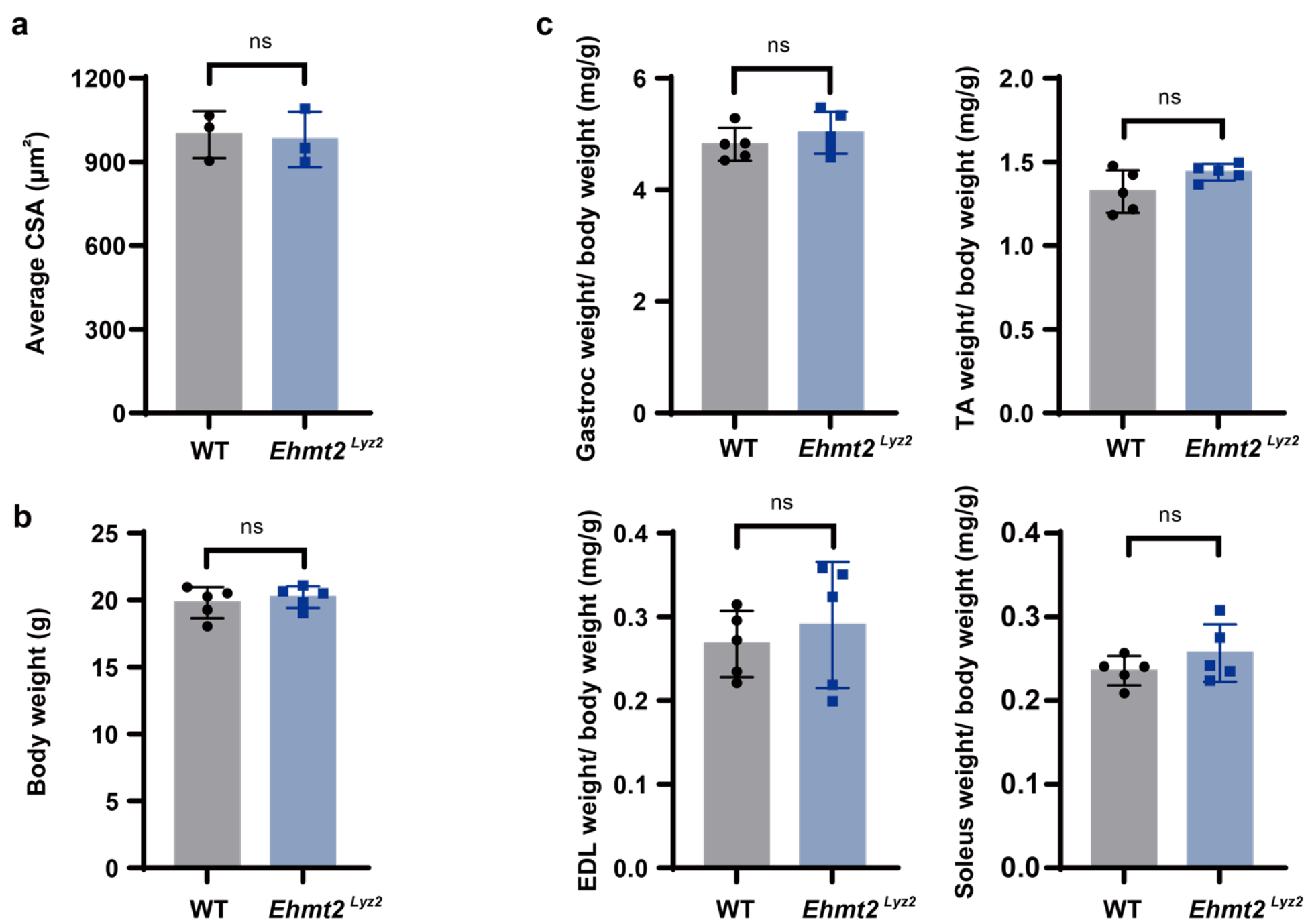

Figure S2 Similar body weights, muscle weights and myofiber morphology between young WT and *Ehmt2*^{Lyz2} KO mice. (a) Average myofiber CSA in the TA muscle of WT and *Ehmt2*^{Lyz2} KO mice. **(b)** Body weights of WT and *Ehmt2*^{Lyz2} KO mice. **(c)** Muscle weights of WT and *Ehmt2*^{Lyz2} KO mice. CSA, cross-sectional area; Gastroc, gastrocnemius; TA, tibialis anterior; EDL, extensor digitorum longus. n = 3 or 5 per group. Data shown as mean ± SD. Mann-Whitney test was used for statistical analysis. ns, no significant difference.

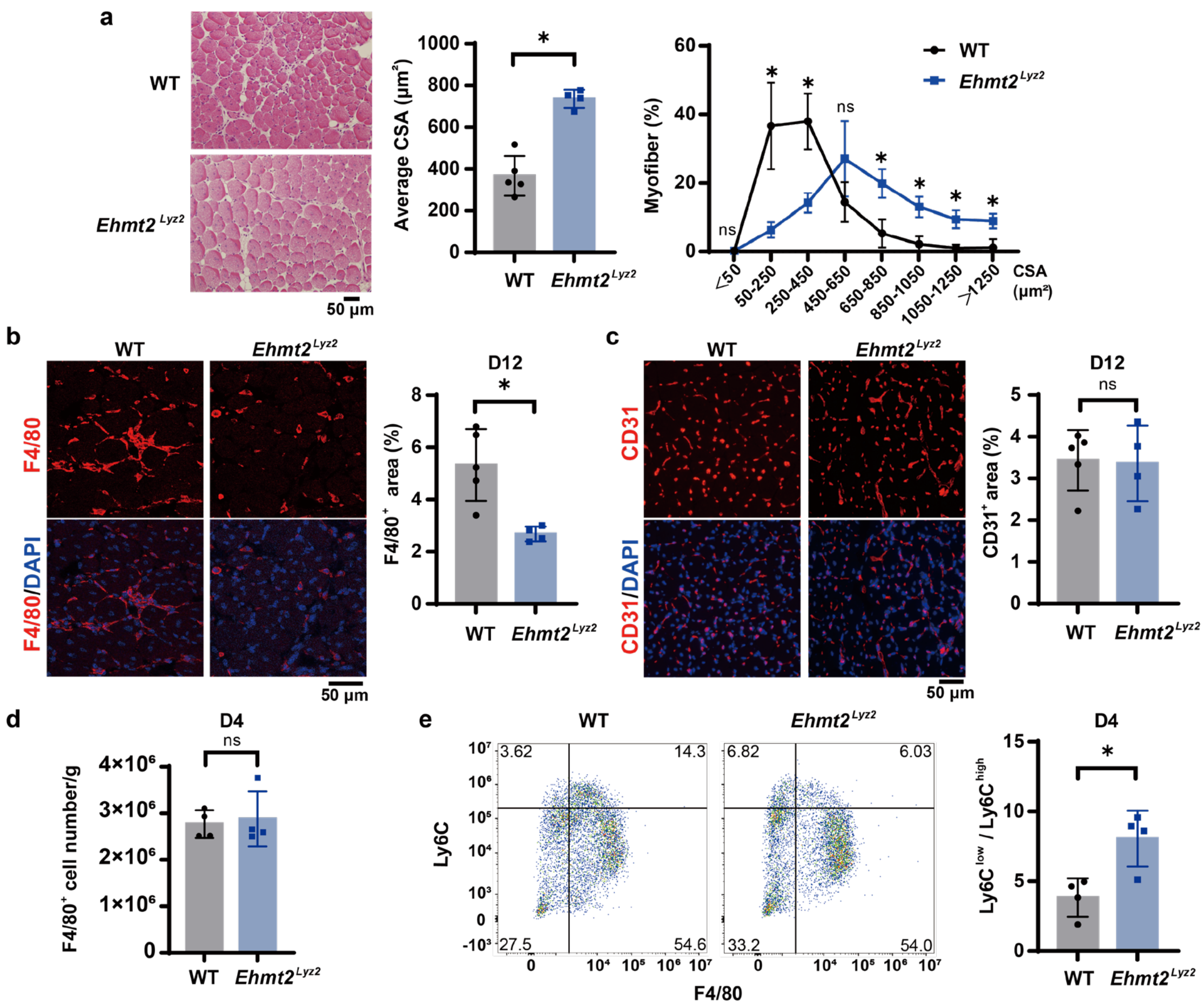

Figure S3 Loss of G9a in myeloid cells accelerates muscle regeneration after HLI -induced muscle injury.

(a) Representative images of H&E staining in the TA muscle of WT and *Ehmt2*^{Lyz2} KO mice (left), with average myofiber CSA (middle) and myofiber frequency distribution (right) at Day 12 post-HLI. **(b)** Representative immunostaining pictures (left) with quantitative analysis (right) for F4/80 in the TA muscle of WT and *Ehmt2*^{Lyz2} KO mice at Day 12 post-HLI. Red, F4/80 staining; blue, DAPI stained nuclei. **(c)** Representative immunostaining pictures (left) with quantitative analysis (right) for CD31 in the TA muscle of WT and *Ehmt2*^{Lyz2} KO mice at Day 12 post-HLI. Red, CD31 staining; blue,

DAPI stained nuclei. **(d)** Number of macrophages ($F4/80^+$) per gram (g) of muscle in the TA muscle of WT and *Ehmt2*^{Lyz2} KO mice at Day 4 post-HLI. **(e)** Representative pseudo-color plot from flow cytometry analysis based on F4/80 and Ly6C (left), with the ratio of anti-inflammatory ($Ly6C^{low}$) to pro-inflammatory macrophages ($Ly6C^{high}$) (right) in the TA muscle of WT and *Ehmt2*^{Lyz2} KO mice at Day 4 post-HLI. TA, tibialis anterior; CSA, cross-sectional area; HLI, hindlimb ischemia. n = 4, 5 per group. Data shown as mean ± SD. Mann-Whitney test was used for statistical analysis. *$P < 0.05$; ns, no significant difference.

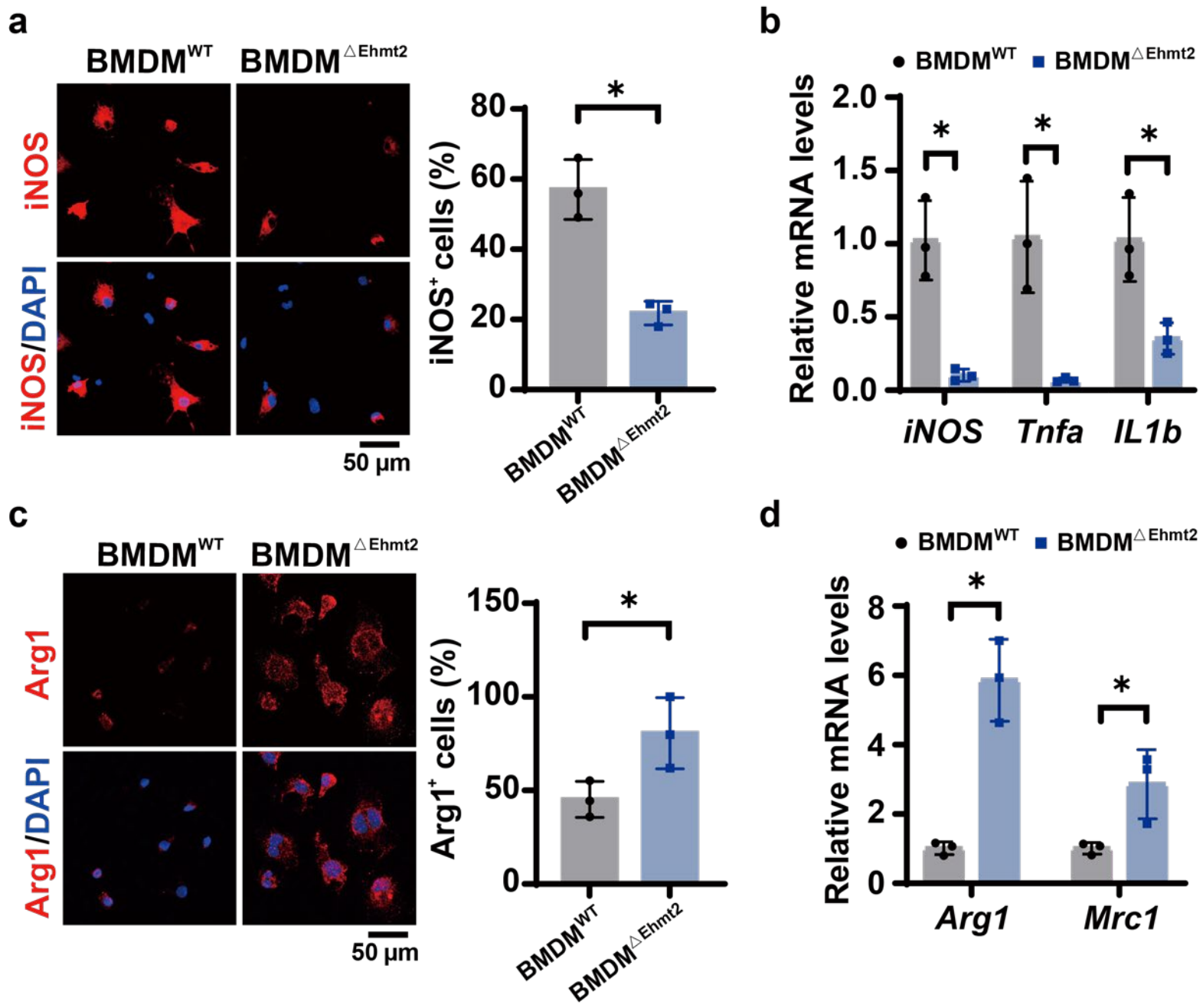

Figure S4 Loss of G9a in myeloid cells affects macrophage polarization. (a, b) Representative immunostaining (left, **a**) with quantitative analysis (right, **a**) of iNOS and transcriptional levels of M1-like macrophage markers (**b**) in the BMDMs isolated from WT or *Ehmt2*^{Lyz2} KO mice (BMDMs^{WT} or BMDMs$^{\Delta Ehmt2}$) treated by LPS plus IFNγ. Red, iNOS staining; blue, DAPI stained nuclei. **(c, d)** Representative immunostaining (left, **c**) with quantitative analysis (right, **c**) of Arg1 and transcriptional levels of M2-like macrophage markers (**d**) in BMDMs^{WT} or BMDMs$^{\Delta Ehmt2}$ treated by IL4. Red, Arg1 staining; blue, DAPI stained nuclei. BMDMs, bone marrow-derived macrophages. n = 3 per group. Data shown as mean ± SD. Mann-Whitney test was used for statistical analysis. *$P < 0.05$.

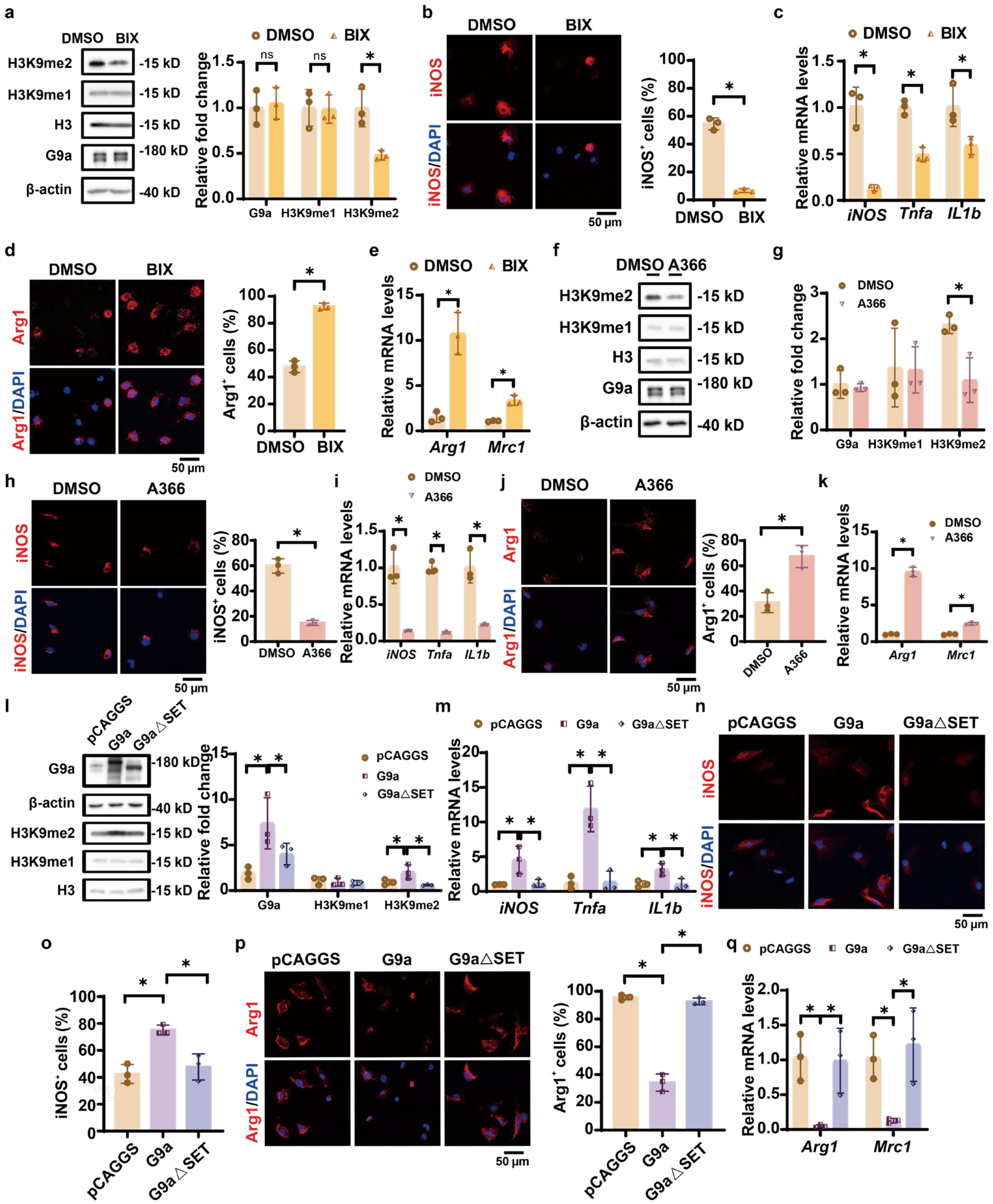

Figure S5 G9a regulates macrophage polarization in a methyltransferase activity-dependent manner. (a) Western blots (left) with quantitative analysis (right) of G9a (normalized to β-actin) and H3K9me1/2 (normalized to histone H3) in BMDMs treated

with or without BIX. **(b, c)** Representative immunostaining (left, **b**) with quantitative analysis (right, **b**) of iNOS and transcriptional levels of M1-like macrophage markers (**c**) in BMDMs treated with or without BIX. Red, iNOS staining; blue, DAPI stained nuclei. **(d, e)** Representative immunostaining (left, **d**) with quantitative analysis (right, **d**) of Arg1 and transcriptional levels of M2-like macrophage markers (**e**) in BMDMs treated with or without BIX. Red, Arg1 staining; blue, DAPI stained nuclei. **(f, g)** Western blots (**f**) with quantitative analysis (**g**) of G9a (normalized to β-actin) and H3K9me1/2 (normalized to histone H3) in BMDMs treated with or without A366. **(h, i)** Representative immunostaining (left, **h**) with quantitative analysis (right, **h**) of iNOS and transcriptional levels of M1-like macrophage markers (**i**) in BMDMs treated with or without A366. Red, iNOS staining; blue, DAPI stained nuclei. **(j, k)** Representative immunostaining (left, **j**) with quantitative analysis (right, **j**) of Arg1 and transcriptional levels of M2-like macrophage markers (**k**) in BMDMs treated with or without A366. Red, Arg1 staining; blue, DAPI stained nuclei. **(l)** Western blots (left) with quantitative analysis (right) of G9a (normalized to β-actin) and H3K9me1/2 (normalized to histone H3) in G9a or G9a△SET overexpressed BMDMs. **(m-o)** Transcriptional levels of M1-like macrophage markers (**m**) and representative immunostaining (**n**) with quantitative analysis (**o**) of iNOS in G9a or G9a△SET overexpressed BMDMs. Red, iNOS staining; blue, DAPI stained nuclei. **(p, q)** Representative immunostaining (left, **p**) with quantitative analysis (right, **p**) and transcriptional levels of M2-like macrophage

markers (**q**) of Arg1 in G9a or G9a△SET overexpressed BMDMs. Red, Arg1 staining; blue, DAPI stained nuclei. BMDMs, bone marrow-derived macrophages. n = 3 per group. Data shown as mean ± SD. Mann-Whitney test was used for statistical analysis of two groups, while Kruskal-Wallis test followed by Mann-Whitney was used for multiple experimental groups. *P < 0.05; ns, no significant difference.

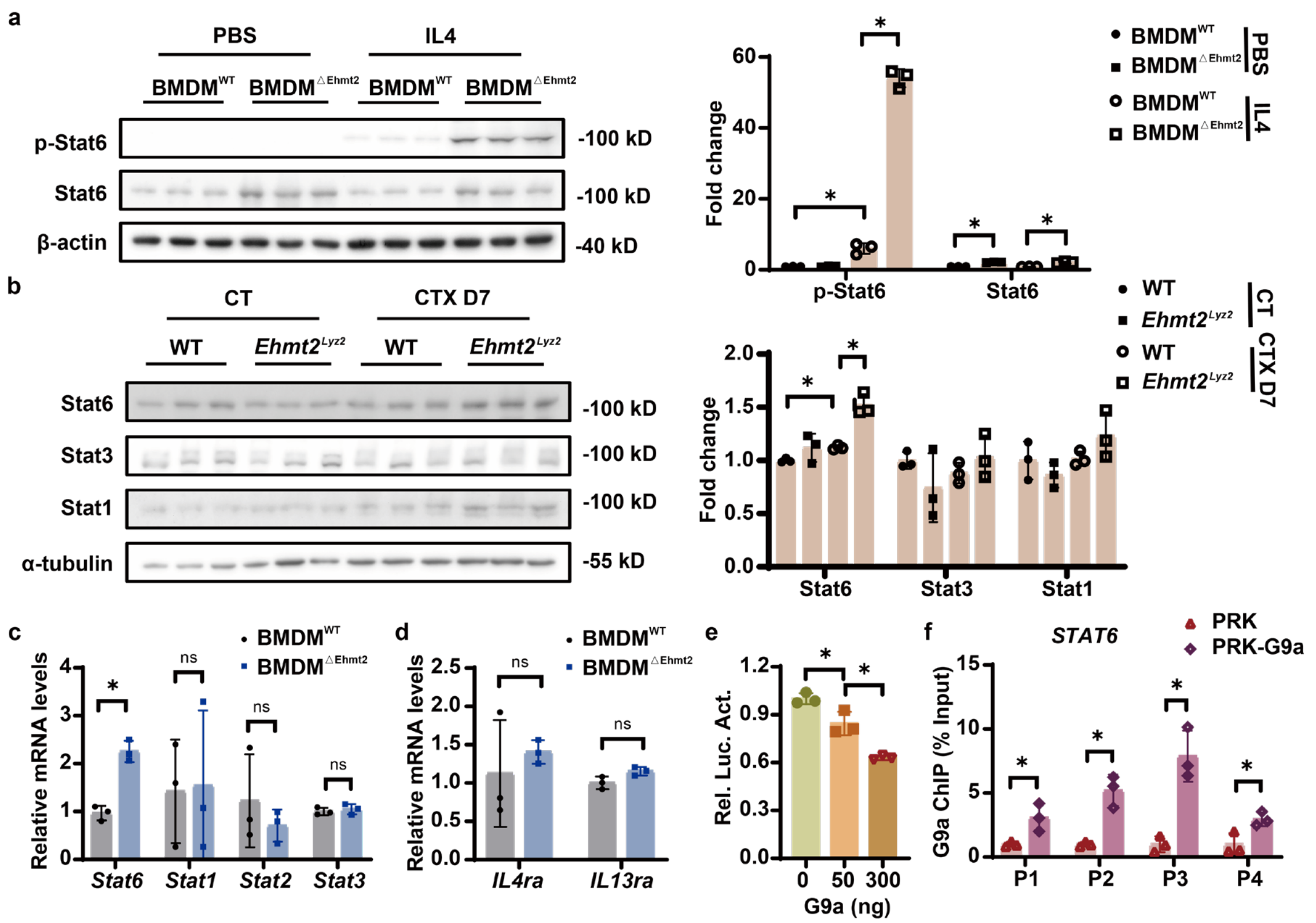

Figure S6 G9a binds to the *STAT6* promoter and inhibits its transcription. (a) Western blots (left) with quantitative analysis (right) of Stat6 and p-Stat6 (normalized to β-actin) in the IL4-treated BMDMs isolated from WT or *Ehmt2*^{Lyz2} KO mice (BMDMs^{WT} or BMDMs$^{\Delta Ehmt2}$). **(b)** Western blots (left) with quantitative analysis (right) of Stat6/3/1 levels (normalized to α-tubulin) in the TA muscle of WT and *Ehmt2*^{Lyz2} KO mice at Day 7 after CTX-injury. **(c, d)** Transcription levels of *Stat6/3/2/1* (**c**) and *IL4ra/IL13ra* (**d**) in BMDMs^{WT} and BMDMs$^{\Delta Ehmt2}$. **(e)** Relative luciferase activity for *STAT6* in G9a overexpressed THP-1 cells. **(f)** G9a binding affinities on the promoter of *STAT6* in G9a overexpressed THP-1 cells. CT, control; BMDMs, bone marrow-derived macrophages; CTX, cardiotoxin; TA, tibialis anterior; n = 3 per group. Data shown as mean ± SD.

Mann-Whitney test was used for statistical analysis of two groups, Kruskal-Wallis test followed by Mann-Whitney test was used for multiple experimental groups. *$P < 0.05$; ns, no significant difference.

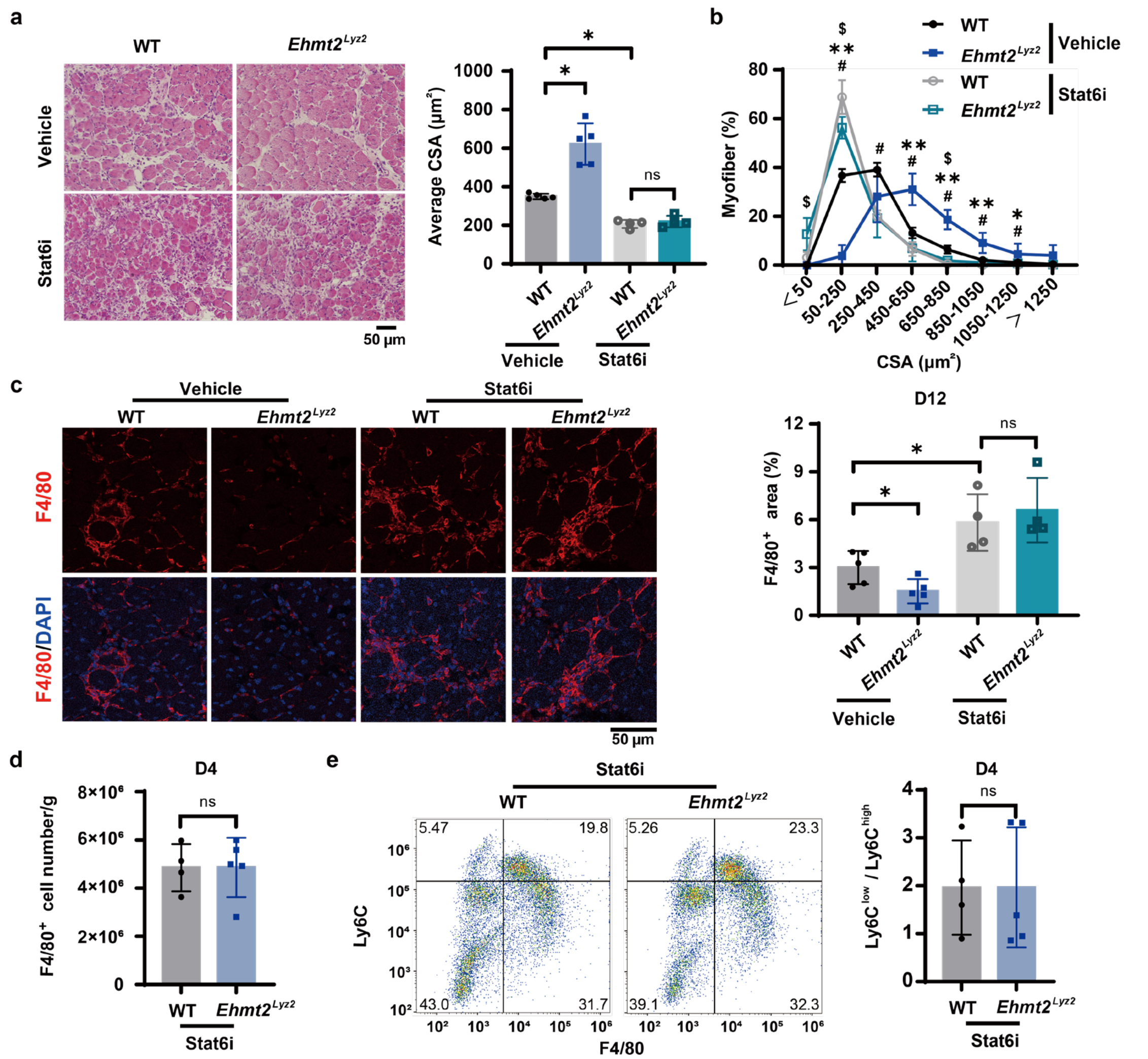

Figure S7 Macrophage G9a inhibits the transition to an anti-inflammatory phenotype *via* Stat6 after HLI-induced muscle injury. (a-c) Representative images of H&E staining (left, **a**) with average myofiber CSA (right, **a**) and its frequency distribution (**b**), as well as representative F4/80 immunostaining (left, **c**) with quantitative analysis (right, **c**) in the TA muscle of WT and $Ehmt2^{Lyz2}$ KO mice treated with or without Stat6i at Day 12 post-HLI. Red, F4/80 staining; blue, DAPI stained nuclei. **(d)** Number of macrophages ($F4/80^+$) per gram (g) of TA muscle of WT and $Ehmt2^{Lyz2}$ KO mice treated

with or without Stat6i at Day 4 post-HLI. **(e)** Representative pseudo-color plot from flow cytometry analysis based on F4/80 and Ly6C (left) and the ratio of anti-inflammatory (Ly6C^{low}) to pro-inflammatory macrophages (Ly6C^{high}) (right) in the TA muscle of WT and *Ehmt2*^{Lyz2} KO mice treated with or without Stat6i at Day 4 post-HLI. TA, tibialis anterior; HLI, hindlimb ischemia; CSA, cross-sectional area; Stat6i, p-Stat6 inhibitor. n = 4-5 per group. Data shown as mean ± SD. Mann-Whitney test was used for statistical analysis of two groups; while Kruskal-Wallis test followed by Mann-Whitney test was used for multiple experimental groups. (**a**, **c-e**): $^{*}P < 0.05$, $^{**}P < 0.01$; ns, no significant difference; (**b**): WT+Vehicle *vs.* *Ehmt2*^{Lyz2}+Vehicle, $^{*}P < 0.05$, $^{**}P < 0.01$; WT+Vehicle *vs.* WT+Stat6i, $^{\#}P < 0.05$; WT+Stat6i *vs.* *Ehmt2*^{Lyz2}+Stat6i, $^{\$}P < 0.05$.

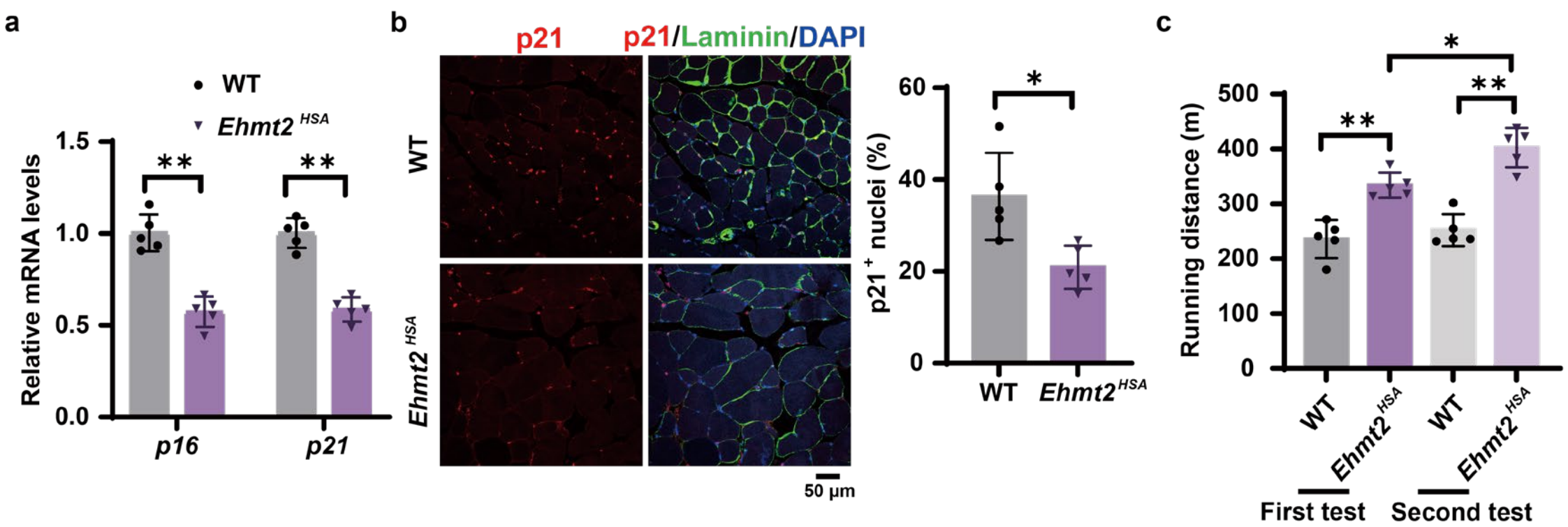

Figure S8 *Ehmt2*^{HSA} KO mice ameliorate aging-induced muscle functional loss. (a) Transcription levels of p16 and p21 in the TA muscle of 18-month-old WT and *Ehmt2*^{HSA} KO mice. **(b)** Representative co-immunostaining pictures of p21 and Laminin (left) with quantitative analysis (right) in the TA muscle of WT and *Ehmt2*^{HSA} KO mice. Red, p21 staining; green, Laminin staining; blue, DAPI stained nuclei. **(c)** Running distance of 18-month-old WT and *Ehmt2*^{HSA} KO mice in the first and second treadmill tests. TA, tibialis anterior. n = 5 per group. Data shown as mean ± SD. Mann-Whitney test was used for statistical analysis of two groups; while Kruskal-Wallis test followed by the Mann-Whitney test was used for multiple experimental groups. *$P < 0.05$, **$P < 0.01$.

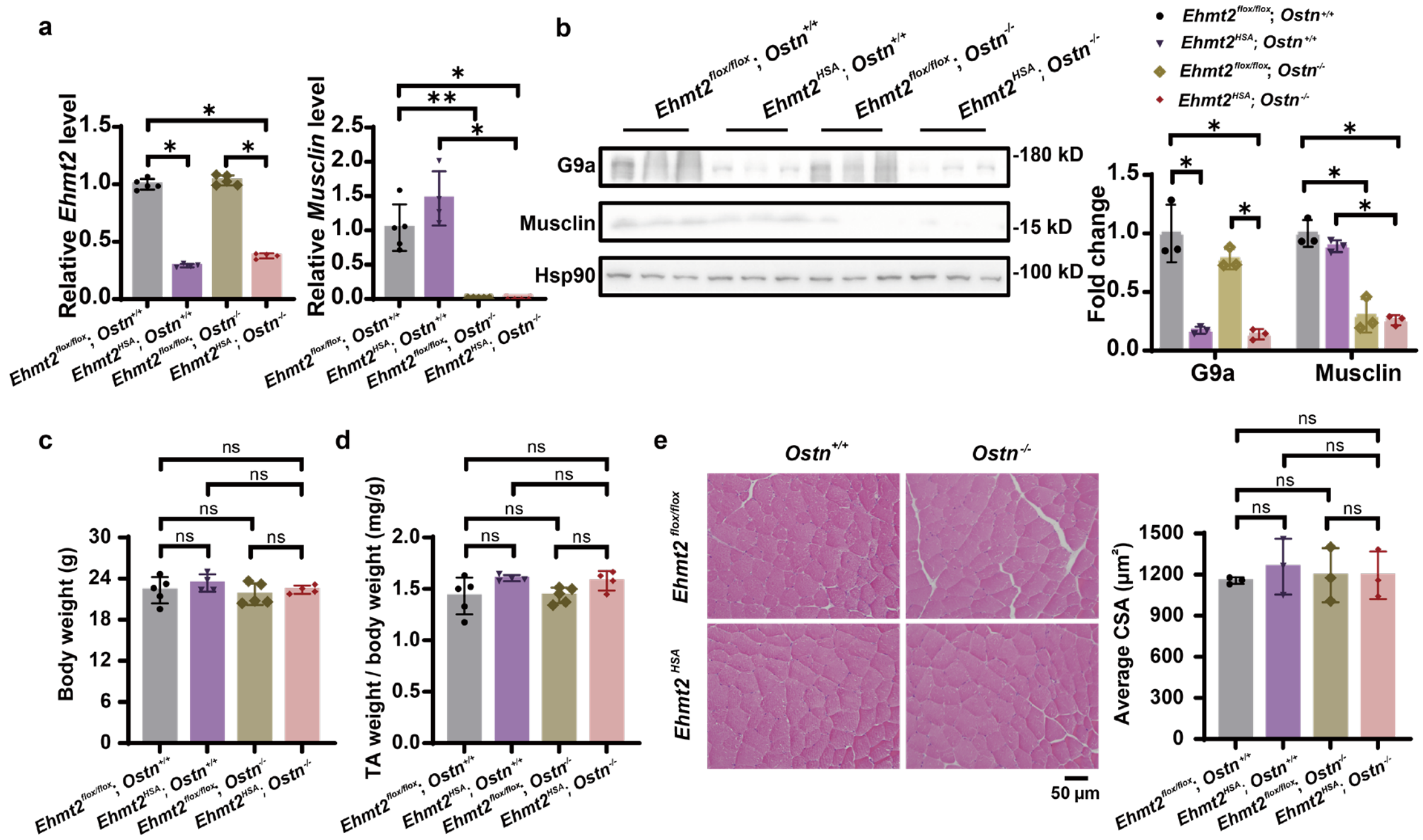

Figure S9 Similar body weights, muscle weights and myofiber morphology between *Ehmt2*^{HSA}; *Ostn*$^{-/-}$ and *Ehmt2*^{HSA}; *Ostn*$^{+/+}$ mice. (a) Transcription levels of *Ehmt2* and *Ostn* in the TA muscle of indicated mouse groups. **(b)** Western blots (left) with quantitative analysis (right) of G9a and Musclin (normalized to Hsp90) in the TA muscle of indicated mouse groups. **(c)** Body weights of indicated mouse groups. **(d)** TA muscle weights of indicated mouse groups. **(e)** Representative images of H&E staining (left) with average myofiber CSA (right) for the TA muscle of indicated mouse groups. TA, tibialis anterior; CSA, cross-sectional area. n = 3-5 per group. Data shown as mean ± SD. Kruskal-Wallis test followed by Mann-Whitney test was used for statistical analysis. **P* < 0.05, ***P* < 0.01; ns, no significant difference.

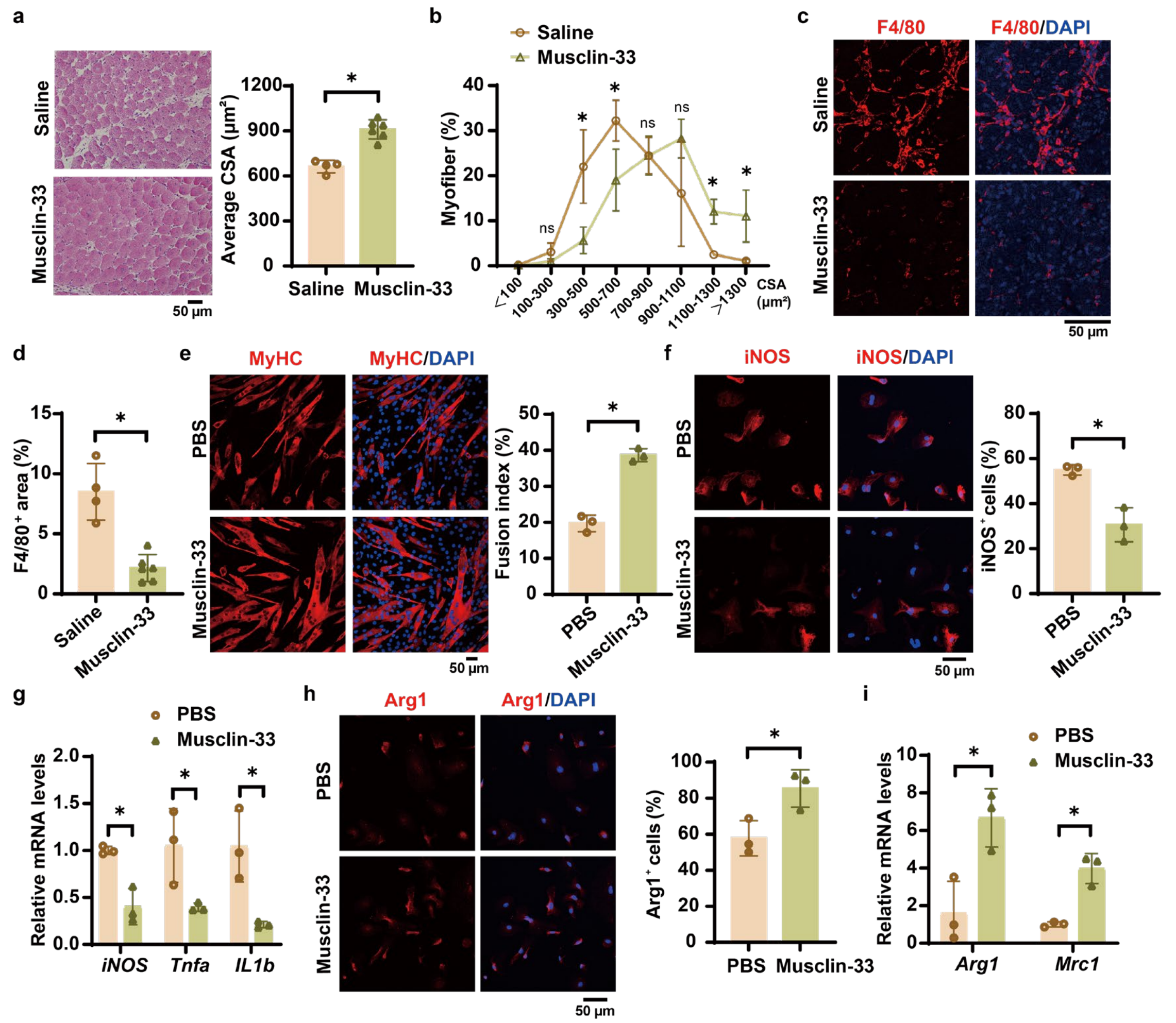

Figure S10 Musclin-33 accelerates muscle regeneration. (a-d) Representative images of H&E staining (left, **a**) with average myofiber CSA (right, **a**) and its frequency distribution (**b**), as well as representative F4/80 immunostaining (**c**) with its quantitative analysis (**d**) in the TA muscle of mice treated with or without Musclin-33 at Day 7 after CTX-injury. Red, F4/80 staining; blue, DAPI stained nuclei. **(e)** Representative immunostaining pictures of MyHC (left) and fusion index analysis (right) in C2C12 cells treated with or without Musclin-33. Red, MyHC; blue, DAPI stained nuclei. **(f, g)** Representative iNOS immunostaining (left, **f**) with quantitative analysis (right, **f**) and

transcriptional levels of M1-like macrophage markers (**g**) in BMDMs treated with or without Musclin-33. Red, iNOS staining; blue, DAPI stained nuclei. **(h, i)** Representative Arg1 immunostaining (left, **h**) with quantitative analysis (right, **h**) and transcriptional levels of M2-like macrophage markers (**i**) in BMDMs treated with or without Musclin-33. Red, Arg1 staining; blue, DAPI stained nuclei. TA, tibialis anterior; CSA, cross-sectional area; BMDMs, bone marrow-derived macrophages; CTX, cardiotoxin; MyHC, Myosin Heavy Chain. n = 3-6 per group. Data shown as mean ± SD. Mann-Whitney test was used for statistical analysis. *$P < 0.05$; ns, no significant difference.

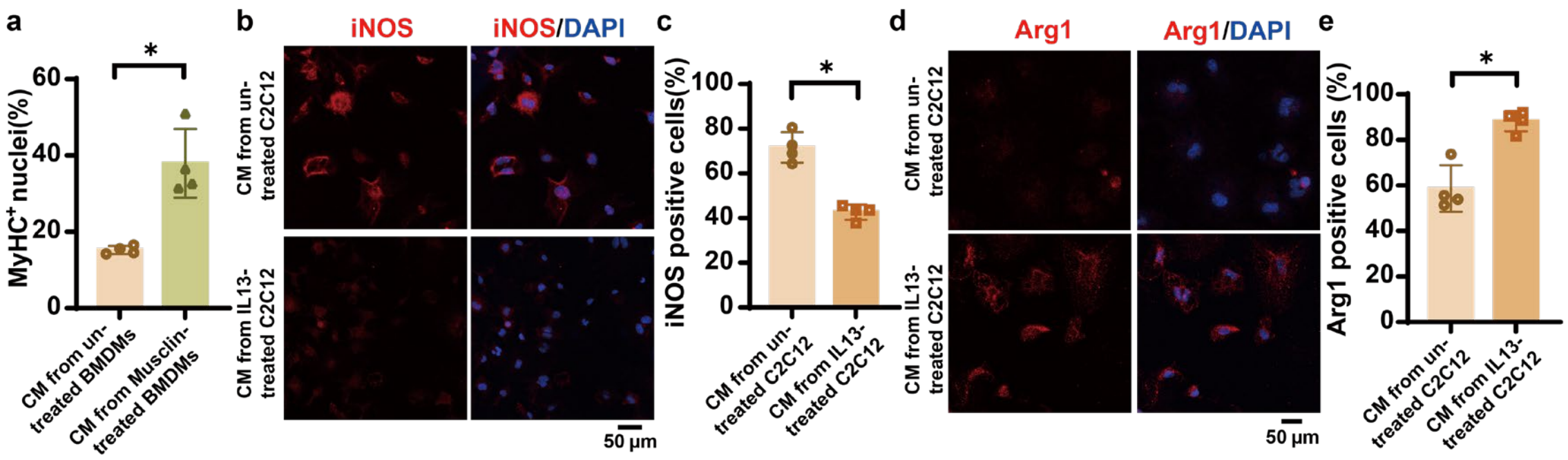

Figure S11 IL13 and Musclin mediate crosstalk between macrophages and myofibers *in vitro*. (a) Fusion index analysis in C2C12 cells treated with conditioned medium from Musclin-treated or un-treated BMDMs (CM from Musclin-treated or un-treated BMDMs). **(b, c)** Representative iNOS immunostaining (**b**) with quantitative analysis (**c**) in BMDMs treated with conditioned medium from IL13-treated or untreated C2C12 cells (CM from IL13-treated or un-treated C2C12). Red, iNOS staining; blue, DAPI stained nuclei. **(d, e)** Representative Arg1 immunostaining (**d**) with quantitative analysis (**e**) in BMDMs treated with conditioned medium from IL13-treated or untreated C2C12 cells. Red, Arg1 staining; blue, DAPI stained nuclei. BMDMs, bone marrow-derived macrophages; MyHC, Myosin Heavy Chain. n = 4 per group. Data shown as mean ± SD. Mann-Whitney test was used for statistical analysis. *$P < 0.05$.
