# Supplementary material for: Deficiency of G9a boosts muscle regeneration through IL13/Musclin-mediated crosstalk between macrophage and myofiber
Source: Cell Death Dis. 2026 Jun 10;17(1):728. doi: 10.1038/s41419-026-08944-2 (PMC13473583; doi:10.1038/s41419-026-08944-2)
Supplement: Supplementary file 2 [file 41419_2026_8944_MOESM2_ESM.pdf]

Full and uncropped western blots

Western blots (WB) and raw data are shown in the following pattern

Up： Western blots shown in Figures

Bottom (Left): Full and uncropped Western blots images merged with membrane with molecular weight markers

Bottom (Right): Full and uncropped Western blots images

Figure 1f

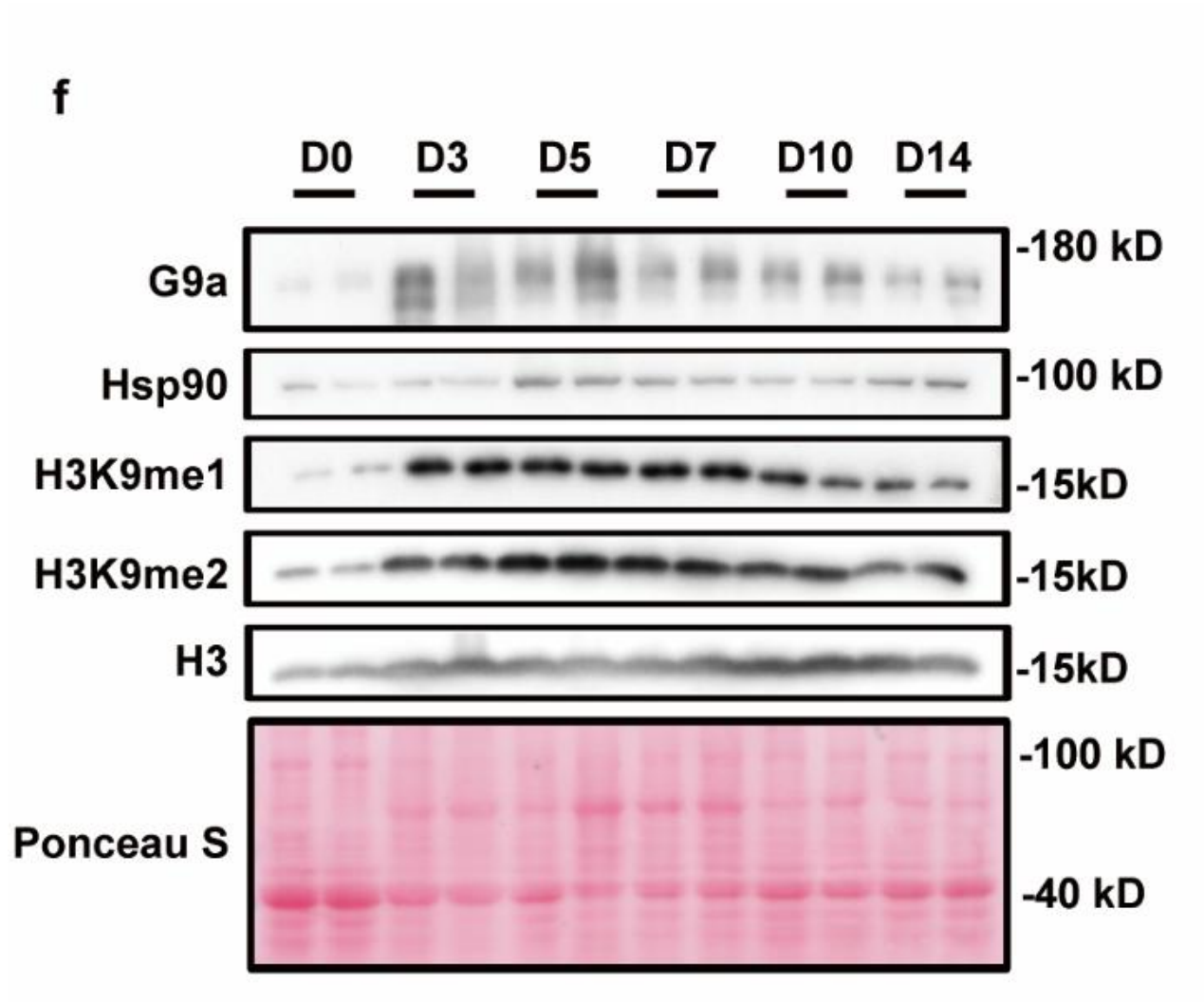
f
D0
D3
D5
D7
D10
D14
G9a
Hsp90
H3K9me1
H3K9me2
H3
Ponceau S
-180 kD
-100 kD
-15kD
-15kD
-15kD
-100 kD
-40 kD

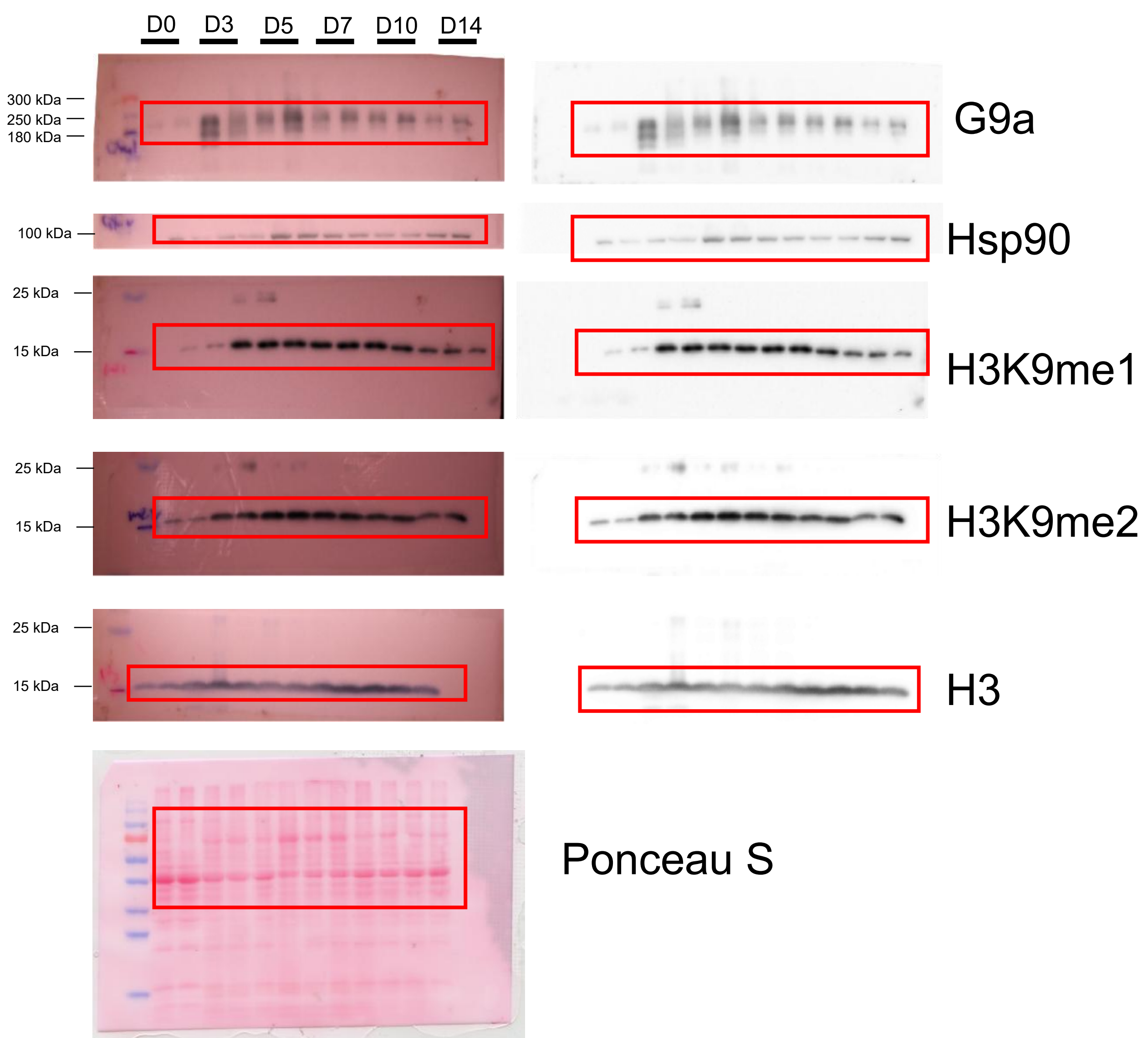
D0
D3
D5
D7
D10
D14
300 kDa
250 kDa
180 kDa
100 kDa
25 kDa
15 kDa
25 kDa
15 kDa
25 kDa
15 kDa
G9a
Hsp90
H3K9me1
H3K9me2
H3
Ponceau S

Figure 2b

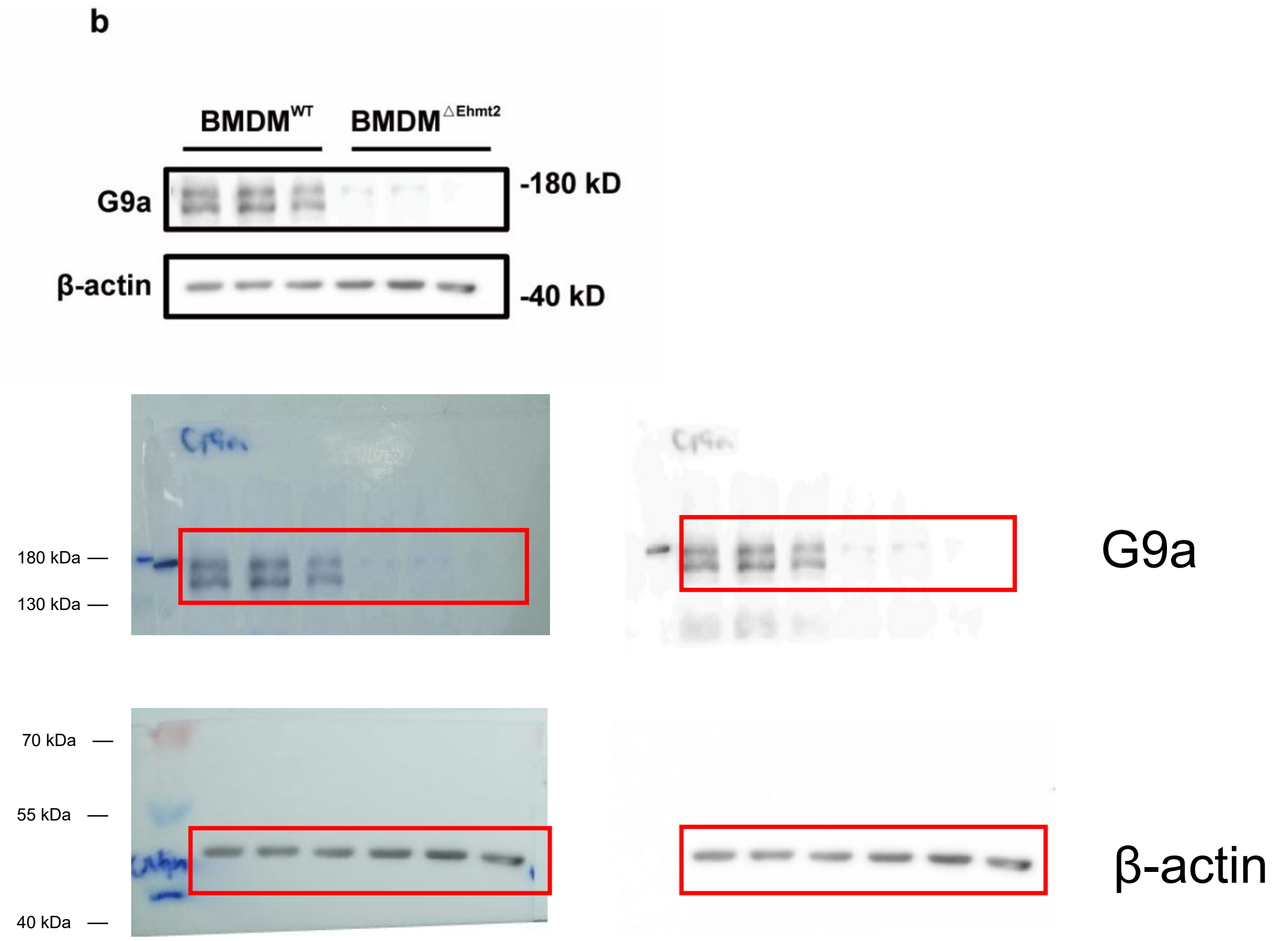
b
BMDM^WT
BMDM^△Ehmt2
G9a
-180 kD
β-actin
-40 kD
180 kDa
130 kDa
70 kDa
55 kDa
40 kDa
G9a
β-actin

Figure 4a

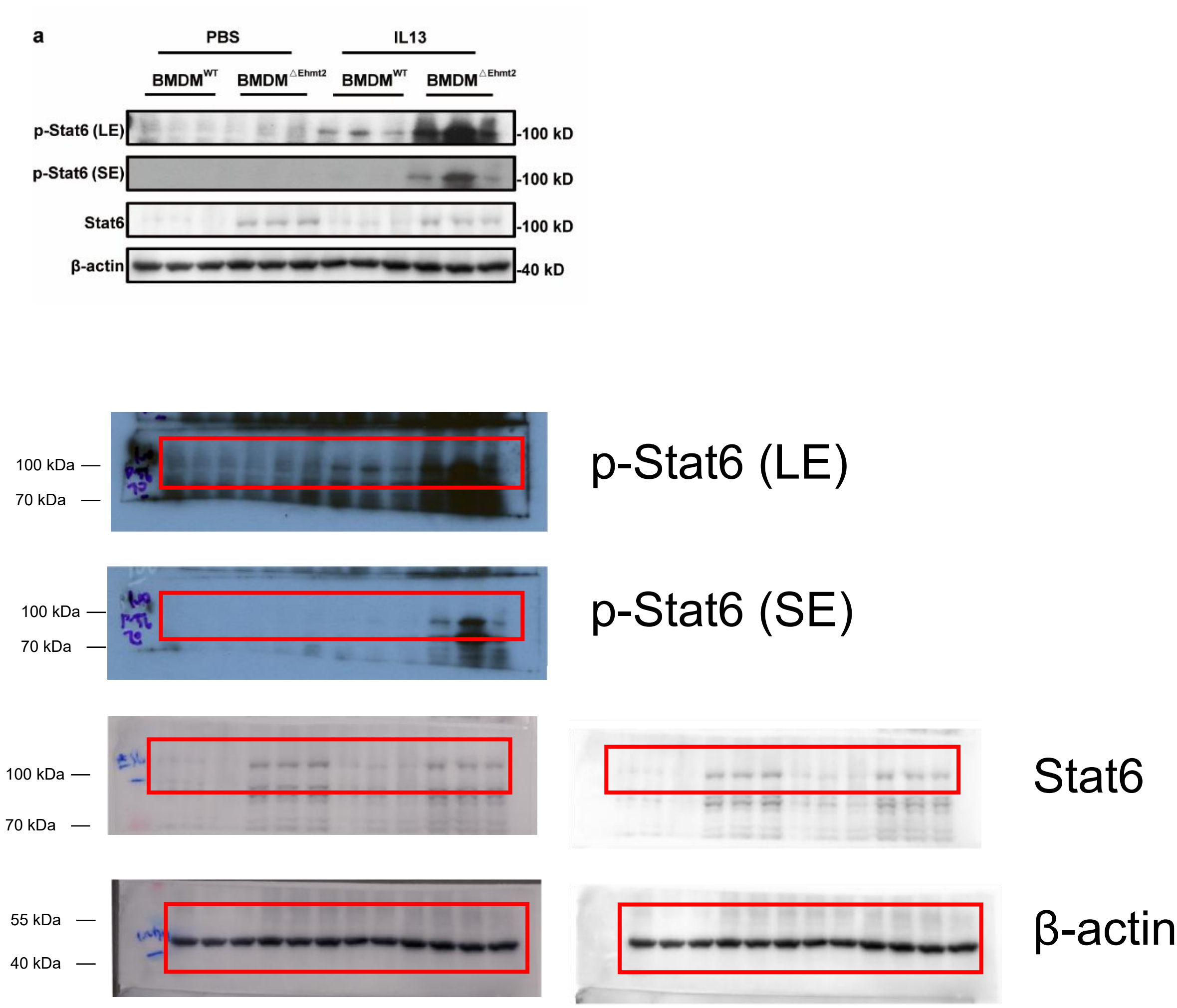
a
PBS
IL13
BMDM^WT
BMDM^△Ehmt2
BMDM^WT
BMDM^△Ehmt2
p-Stat6 (LE)
p-Stat6 (SE)
Stat6
β-actin
-100 kD
-100 kD
-100 kD
-40 kD
100 kDa
70 kDa
p-Stat6 (LE)
100 kDa
70 kDa
p-Stat6 (SE)
100 kDa
70 kDa
Stat6
55 kDa
40 kDa
β-actin

Figure 4c

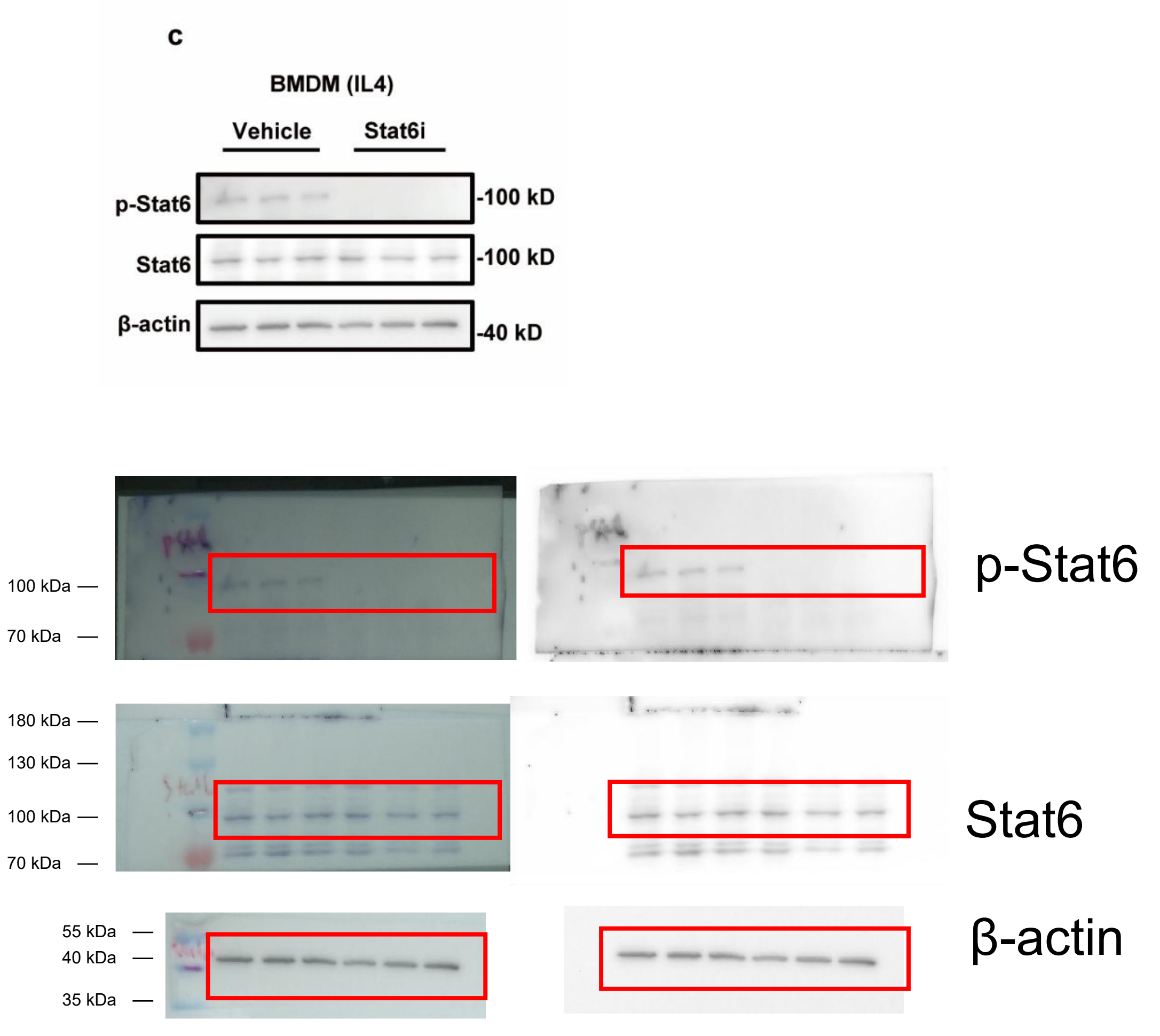

c
BMDM (IL4)
Vehicle
Stat6i
p-Stat6
Stat6
β-actin
-100 kD
-100 kD
-40 kD
100 kDa
70 kDa
180 kDa
130 kDa
100 kDa
70 kDa
55 kDa
40 kDa
35 kDa
p-Stat6
Stat6
β-actin

Figure 6e

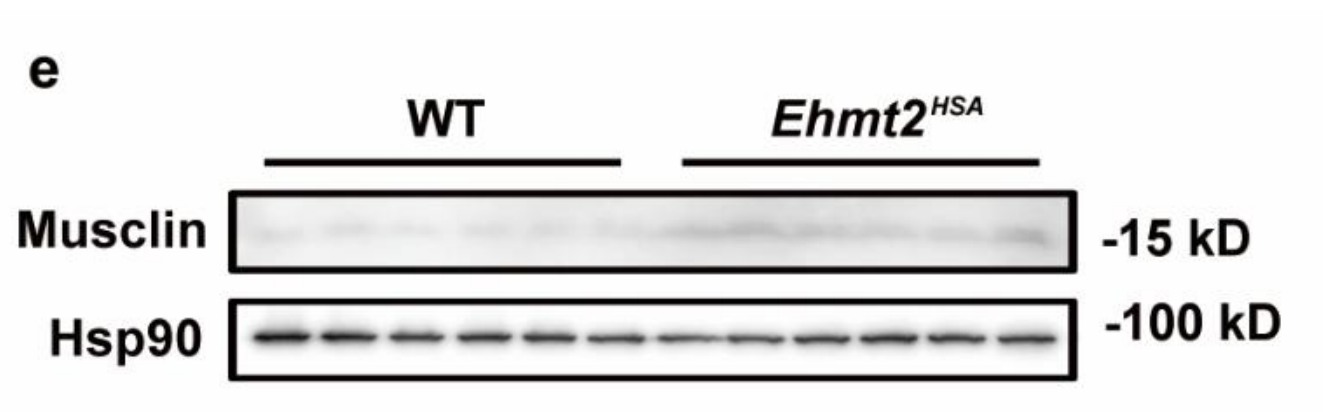
e
WT
Ehmt2HSA
Musclin
Hsp90
-15 kD
-100 kD

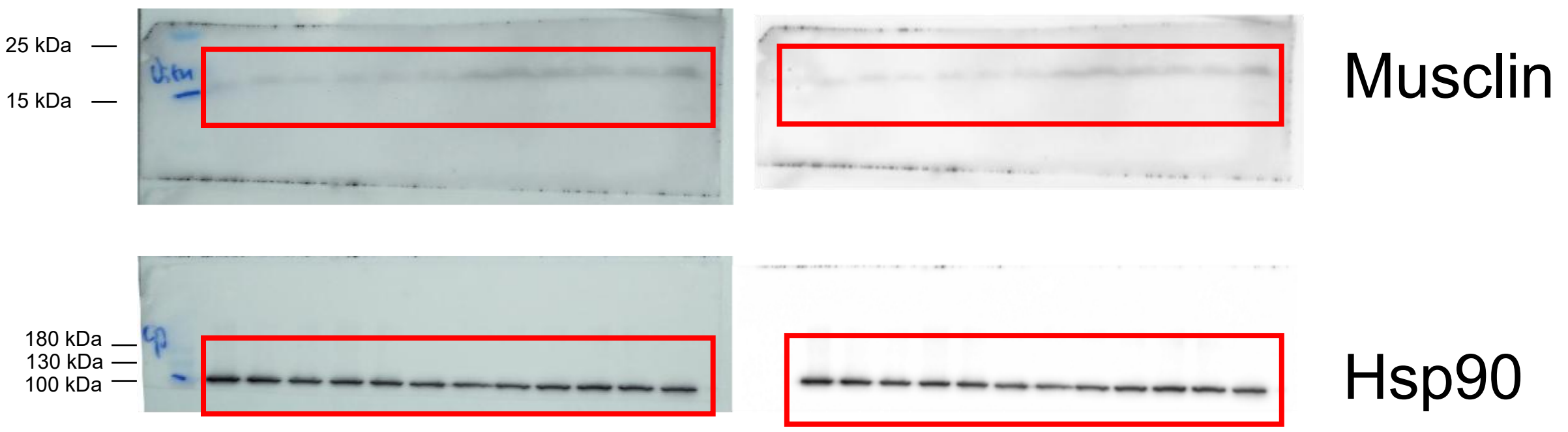
25 kDa
15 kDa
180 kDa
130 kDa
100 kDa
Musclin
Hsp90

Figure S1

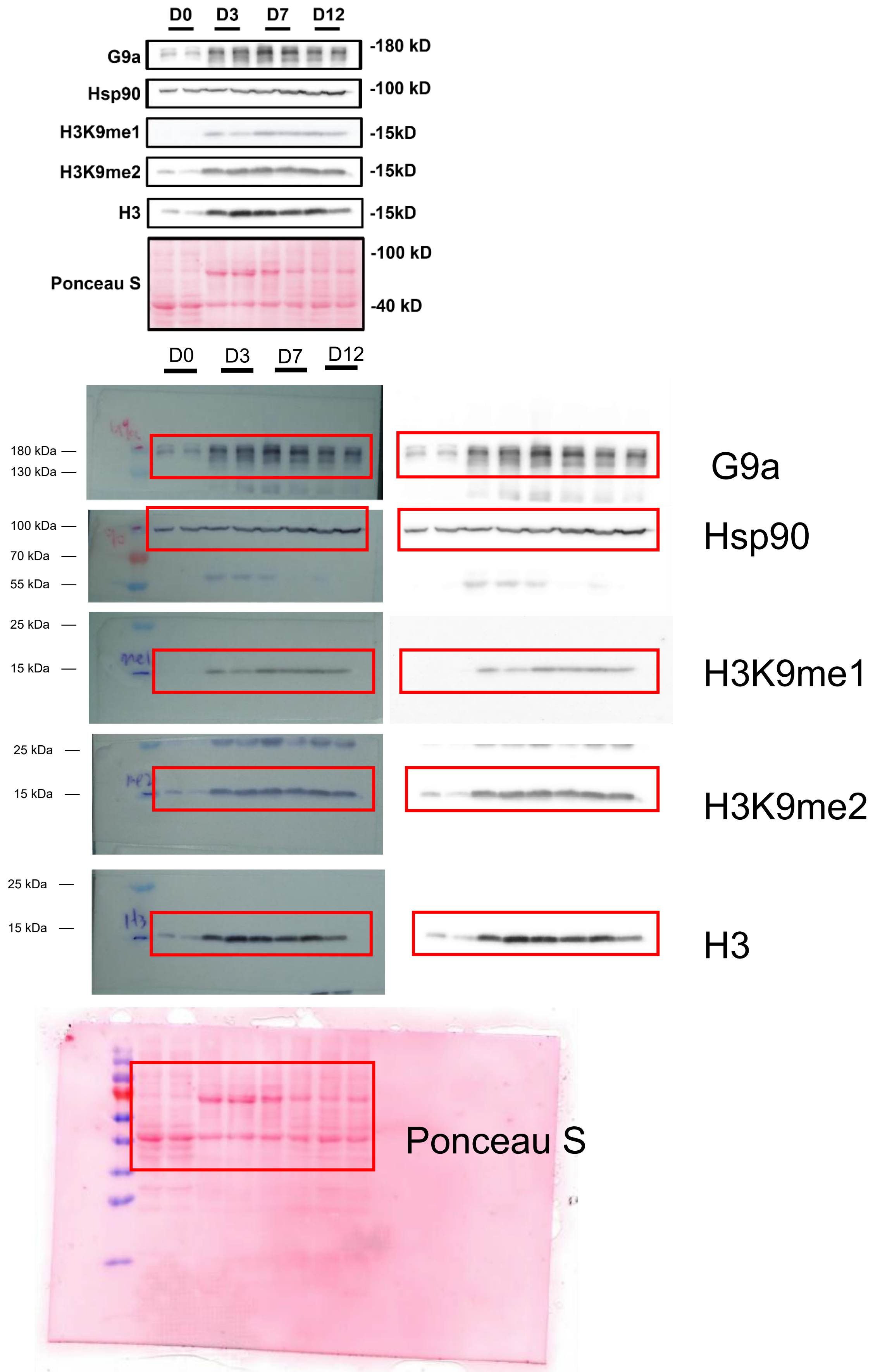

D0
D3
D7
D12
G9a
Hsp90
H3K9me1
H3K9me2
H3
Ponceau S
-180 kD
-100 kD
-15kD
-15kD
-15kD
-100 kD
-40 kD
D0
D3
D7
D12
180 kDa
130 kDa
100 kDa
70 kDa
55 kDa
25 kDa
15 kDa
25 kDa
15 kDa
25 kDa
15 kDa
G9a
Hsp90
H3K9me1
H3K9me2
H3
Ponceau S

Figure S5a

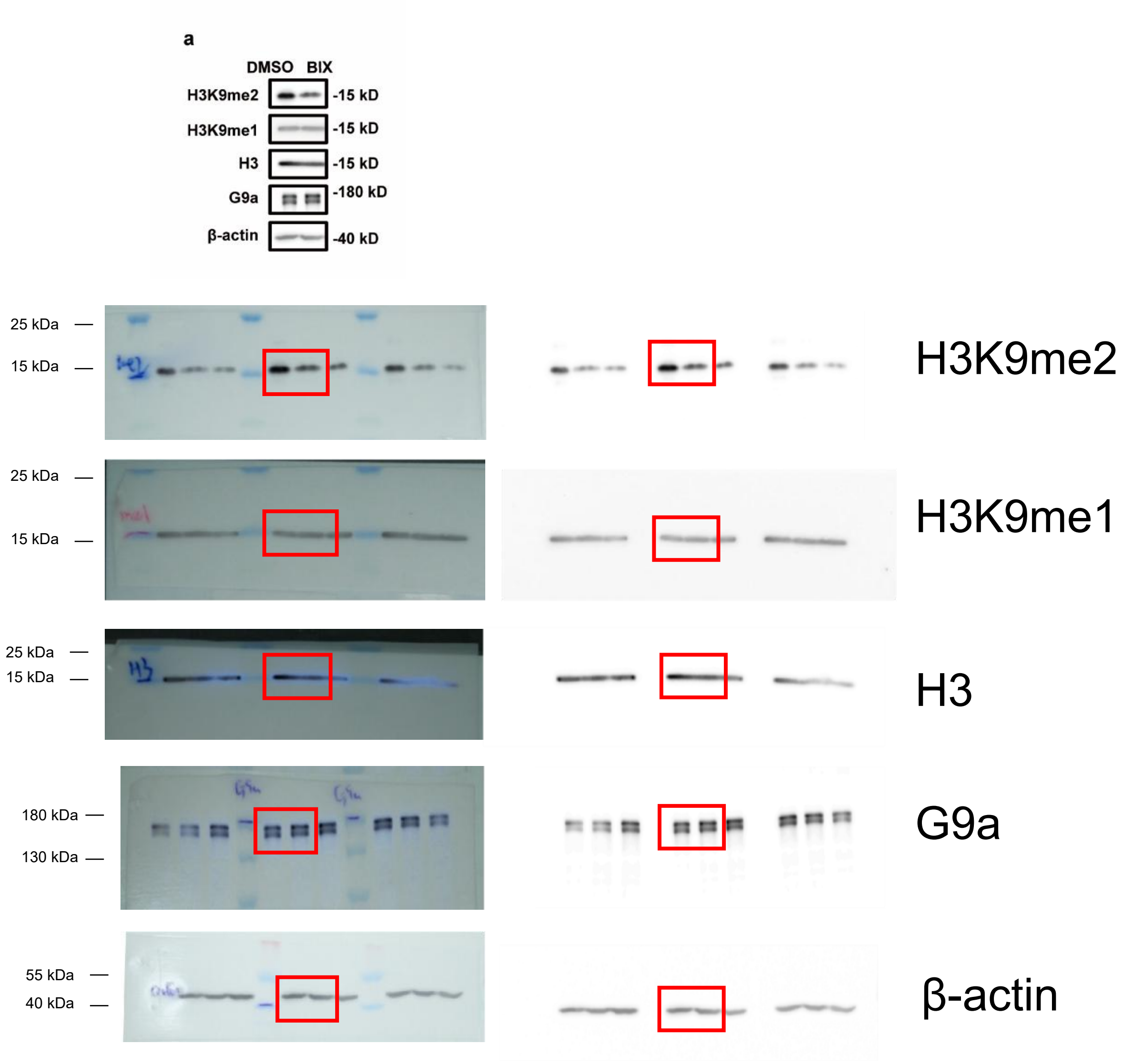
a
DMSO
BIX
H3K9me2
-15 kD
H3K9me1
-15 kD
H3
-15 kD
G9a
-180 kD
β-actin
-40 kD
25 kDa
15 kDa
H3K9me2
25 kDa
15 kDa
H3K9me1
25 kDa
15 kDa
H3
180 kDa
130 kDa
G9a
55 kDa
40 kDa
β-actin

Figure S5f

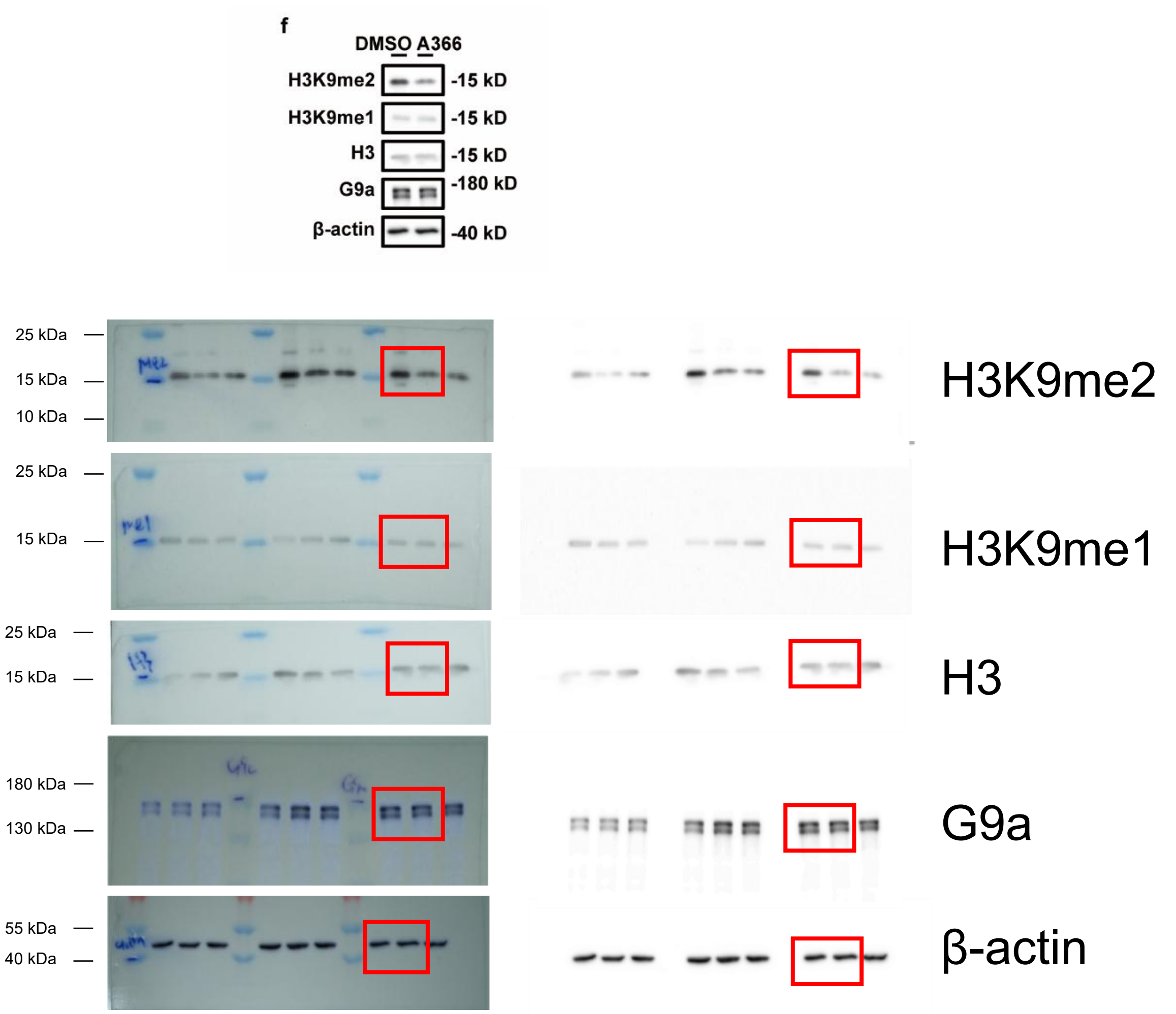
f
DMSO A366
H3K9me2
H3K9me1
H3
G9a
β-actin
-15 kD
-15 kD
-15 kD
-180 kD
-40 kD
25 kDa
15 kDa
10 kDa
25 kDa
15 kDa
25 kDa
15 kDa
180 kDa
130 kDa
55 kDa
40 kDa
H3K9me2
H3K9me1
H3
G9a
β-actin

Figure S5I

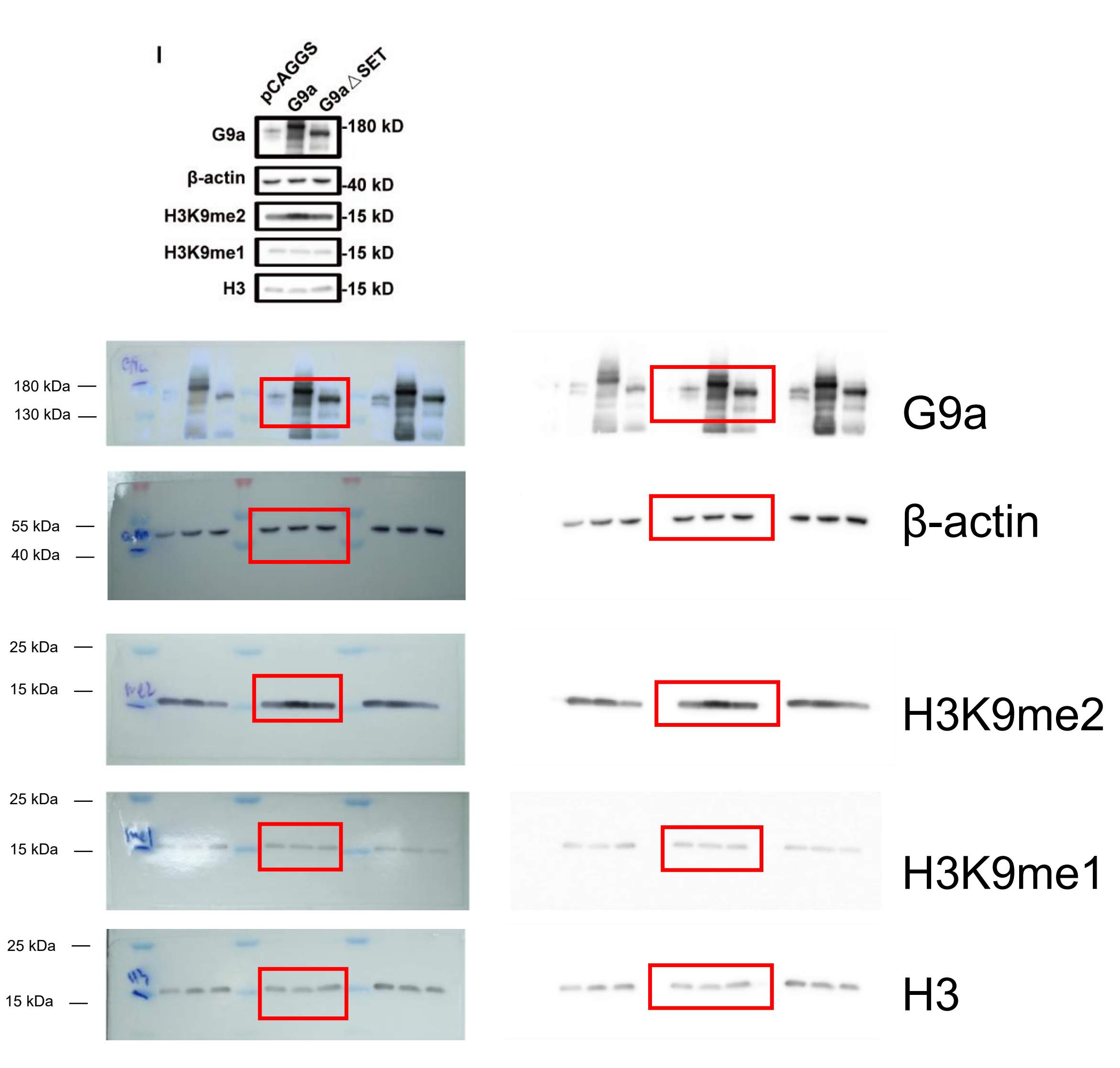

I
pCAGGS
G9a
G9a△SET
G9a
β-actin
H3K9me2
H3K9me1
H3
180 kD
40 kD
15 kD
15 kD
15 kD
180 kDa
130 kDa
55 kDa
40 kDa
25 kDa
15 kDa
25 kDa
15 kDa
25 kDa
15 kDa
G9a
β-actin
H3K9me2
H3K9me1
H3

Figure S6a

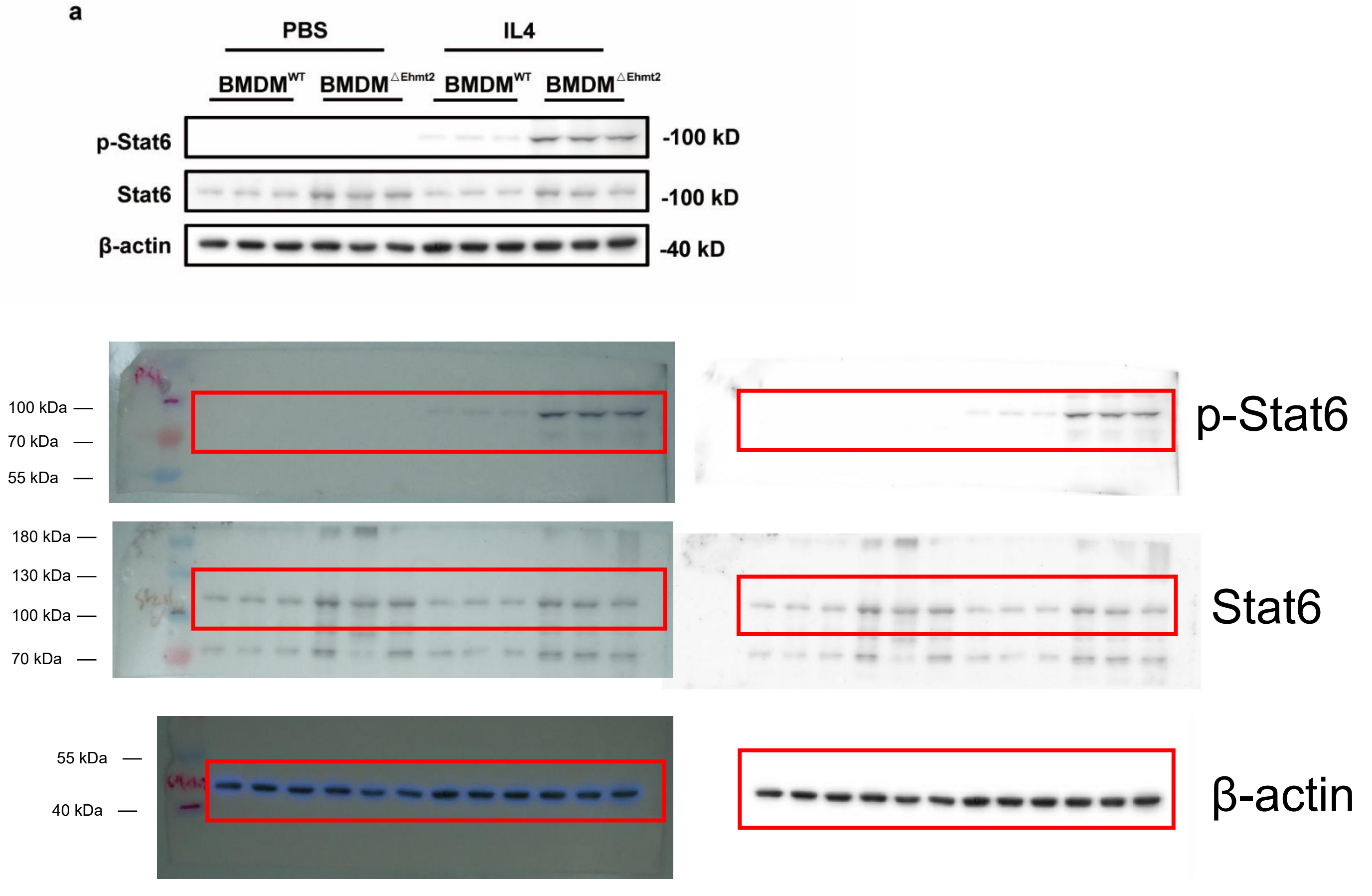
a
PBS
IL4
BMDM^WT
BMDM^△Ehmt2
BMDM^WT
BMDM^△Ehmt2
p-Stat6
Stat6
β-actin
-100 kD
-100 kD
-40 kD
100 kDa
70 kDa
55 kDa
180 kDa
130 kDa
100 kDa
70 kDa
55 kDa
40 kDa
p-Stat6
Stat6
β-actin

Figure S6b

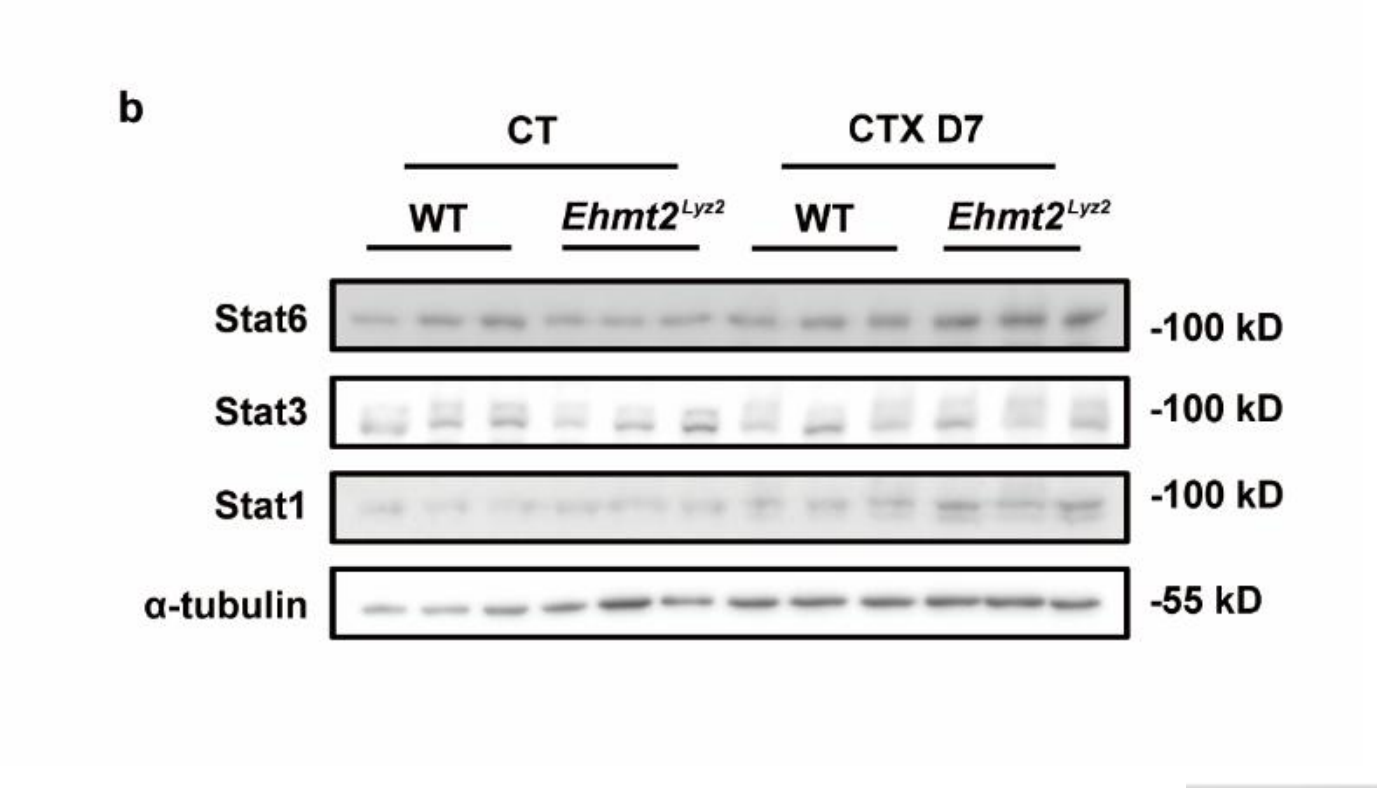
b
CT
CTX D7
WT
Ehmt2Lyz2
WT
Ehmt2Lyz2
Stat6
Stat3
Stat1
α-tubulin
-100 kD
-100 kD
-100 kD
-55 kD

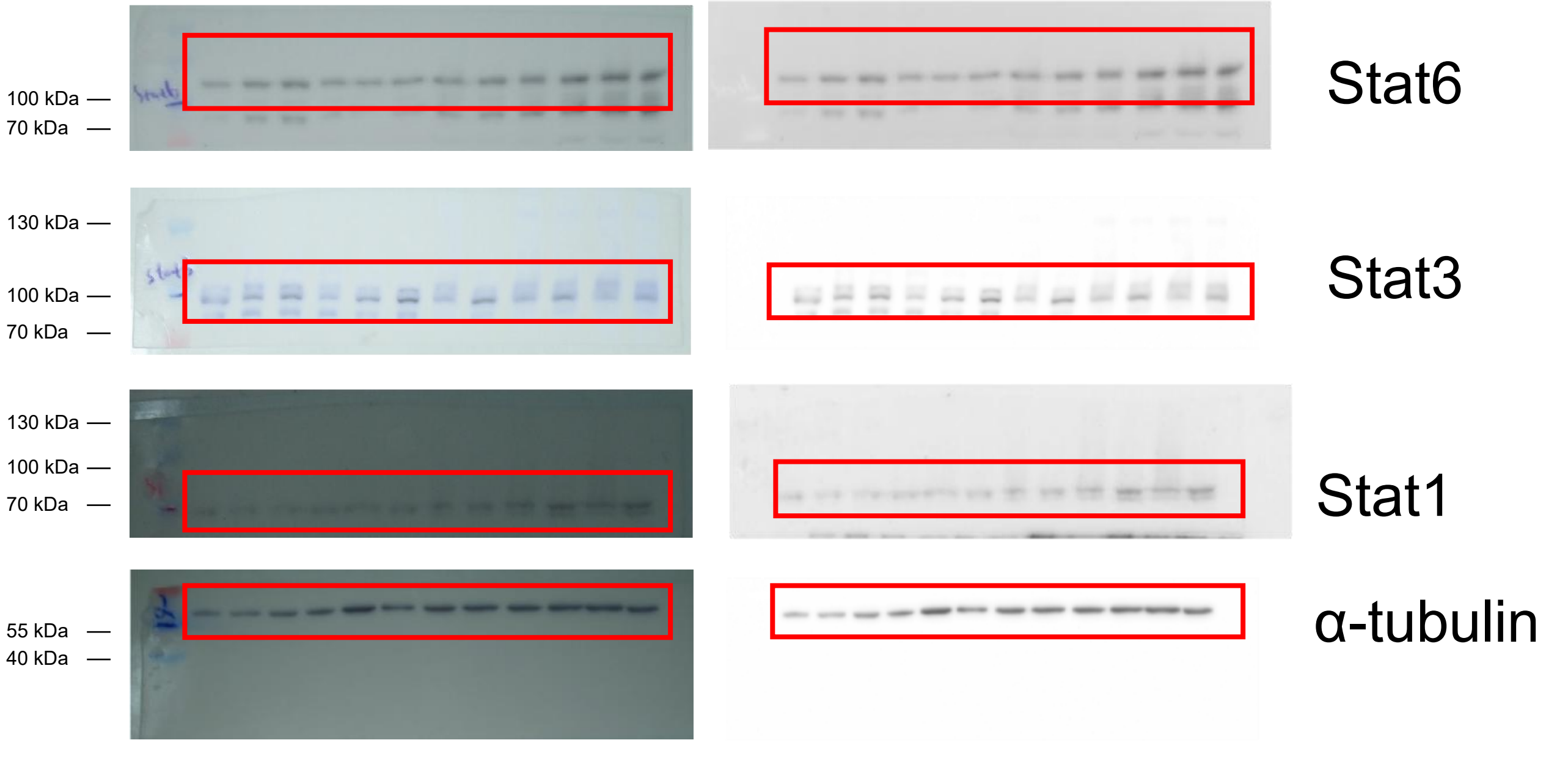
100 kDa
70 kDa
130 kDa
100 kDa
70 kDa
130 kDa
100 kDa
70 kDa
55 kDa
40 kDa
Stat6
Stat3
Stat1
α-tubulin

Figure S9b

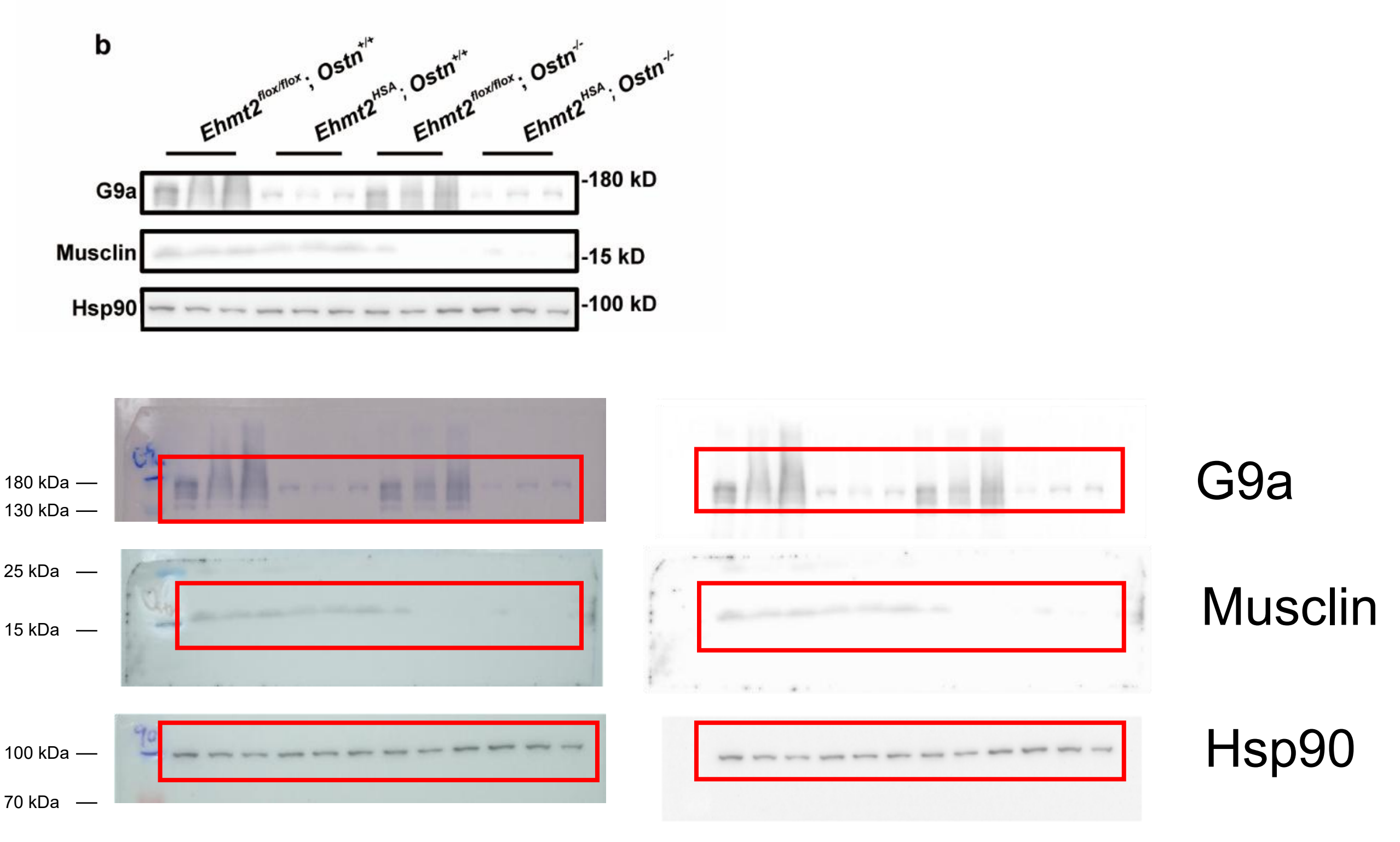
b
Ehmt2flox/flox; Ostn+/+
Ehmt2HSA; Ostn+/+
Ehmt2flox/flox; Ostn-/-
Ehmt2HSA; Ostn-/-
G9a
Musclin
Hsp90
-180 kD
-15 kD
-100 kD
180 kDa
130 kDa
25 kDa
15 kDa
100 kDa
70 kDa
G9a
Musclin
Hsp90
